# Stress-Induced PARP Activation Mediates Recruitment of *Drosophila* Mi-2 to Promote Heat Shock Gene Expression

**DOI:** 10.1371/journal.pgen.1002206

**Published:** 2011-07-28

**Authors:** Magdalena Murawska, Markus Hassler, Renate Renkawitz-Pohl, Andreas Ladurner, Alexander Brehm

**Affiliations:** 1 Institute of Tumor Research and Molecular Biology, Philipps University, Marburg, Germany; 2 Genome Biology and Structural and Computational Biology Unit, European Molecular Biology Laboratory, Heidelberg, Germany; 3 Developmental Biology, Philipps University, Marburg, Germany; 4 Department of Physiological Chemistry, Adolf-Butenandt-Institute, Ludwig-Maximilians University, Munich, Germany; Max-Planck-Institute of Immunobiology, Germany

## Abstract

Eukaryotic cells respond to genomic and environmental stresses, such as DNA damage and heat shock (HS), with the synthesis of poly-[ADP-ribose] (PAR) at specific chromatin regions, such as DNA breaks or HS genes, by PAR polymerases (PARP). Little is known about the role of this modification during cellular stress responses. We show here that the nucleosome remodeler dMi-2 is recruited to active HS genes in a PARP–dependent manner. dMi-2 binds PAR suggesting that this physical interaction is important for recruitment. Indeed, a dMi-2 mutant unable to bind PAR does not localise to active HS loci *in vivo.* We have identified several dMi-2 regions which bind PAR independently *in vitro*, including the chromodomains and regions near the N-terminus containing motifs rich in K and R residues. Moreover, upon HS gene activation, dMi-2 associates with nascent HS gene transcripts, and its catalytic activity is required for efficient transcription and co-transcriptional RNA processing. RNA and PAR compete for dMi-2 binding *in vitro*, suggesting a two step process for dMi-2 association with active HS genes: initial recruitment to the locus via PAR interaction, followed by binding to nascent RNA transcripts. We suggest that stress-induced chromatin PARylation serves to rapidly attract factors that are required for an efficient and timely transcriptional response.

## Author Summary

Cells respond to elevated temperatures with the rapid activation of heat shock genes to ensure cellular survival. Heat shock gene activation involves the synthesis of poly-[ADP-ribose] (PAR) at heat shock loci, the opening of chromatin structure, and the coordinated recruitment of transcription factors and chromatin regulators RNA polymerase II and components of the RNA processing machinery. The molecular roles of PAR and and ATP-dependent chromatin remodelers in heat shock gene activation are not clear. We show here that the chromatin remodeler dMi-2 is recruited to *Drosophila* heat shock genes in a PAR–dependent manner. We provide evidence that recruitment involves direct binding of dMi-2 to PAR polymers and identify novel PAR sensing regions in the dMi-2 protein, including the chromodomains and a series of motifs rich in K and R residues. Upon HS gene activation, dMi-2 associates with nascent transcripts. In addition, we find that dMi-2 and its catalytic activity are important for heat shock gene activation and co-transcriptional RNA processing efficiency. Our study uncovers a novel role of PAR during heat shock gene activation and establishes an unanticipated link between chromatin remodeler activity and RNA processing.

## Introduction

The activity of eukaryotic genomes is regulated by dynamic changes in chromatin structure. A multitude of nucleosome remodeling enzymes, histone modifying activities and chromatin binding proteins cooperate to establish, maintain and reprogram chromatin structures that determine genome activity.

*Drosophila* heat shock (HS) genes provide a textbook example of how dramatic changes in the organismal and cellular environment affect chromatin structure in a manner that promotes transcriptional activation of genes coding for molecular chaperones required during the HS response. Upon temperature shift, the HS loci of polytene chromosomes form transcriptionally active “puffs”. This rapid chromatin decondensation correlates with a strong decrease in nucleosome density [1]. Puff formation can be uncoupled from transcription and much of the nucleosome loss at the *hsp70* gene occurs prior to the first round of transcription [1], [2]. Recently, heat shock factor (HSF), GAGA factor and poly-[ADP-ribose] polymerase (PARP) have been shown to be required for the rapid removal of nucleosomes upon activation of the *hsp70* gene [1]. In addition, HS puffs accumulate PARylated proteins and puff formation depends on PARP activity [3]. The mechanisms underlying PARP action during HS gene activation are not clear. It has been suggested that PARylation may be removing proteins, including histones - which are themselves a good PARP substrate - thereby promoting chromatin opening [1]. The accumulation of PARylated proteins at HS loci has recently been proposed to build up a “transcription compartment” which hinders the diffusion of proteins into and out of the compartment, thus favouring factor recycling [4]. In addition to histone displacement and transcription compartment formation at HS genes, recent evidence suggests that PARylation could also act as a signaling scaffold for the recruitment of PAR-sensing factors during DNA damage. In mammals PARylation at DNA damage sites can mediate the recruitment of several ATP-dependent nucleosome remodeling enzymes [5]–[10]. Here we sought to address whether and how nucleosome remodelers may be recruited to PARP activation sites upon environmental stresses other than DNA damage. We have investigated a paradigm of environmental stress, the activation of HS loci in *Drosophila* and have analyzed the mechanism through which the nucleosome remodeler dMi-2 is recruited to HS genes.

Mi-2 (CHD3/CHD4) is a conserved ATP-dependent nucleosome remodeler. In both vertebrates and invertebrates, it is a subunit of *Nucleosome Remodeling and Deacetylation* (NuRD) complexes. NuRD complexes repress cell type specific genes during differentiation [11]–[13]. dMi-2 is also a subunit of the *Drosophila-specific Mep-1 complex* (dMec) which represses neuron-specific genes during differentiation of the peripheral nervous system [12], [14].

Mi-2 containing complexes lack subunits with sequence-specific DNA binding activity. Two main mechanisms for their recruitment to chromatin have been suggested. First, NuRD complexes contain subunits with *methylated DNA binding domains* (MBD) which direct NuRD to methylated DNA [15], [16]. This is unlikely to be a major recruitment mechanism for *Drosophila* Mi-2 complexes, however, given the low and transient levels of DNA methylation in this organism [17]. A second mode of Mi-2 recruitment involves interactions with DNA bound transcription factors [11], [12], [14], [18]–[22]. In addition, SUMOylation of transcription factors can increase their affinity for Mi-2 complexes [21], [22].

Despite its well established role in repression, dMi-2 localises to actively transcribed chromosome regions suggesting an unexpected potential function of dMi-2 in transcription [23]. Here we sought to establish how dMi-2 is recruited to actively transcribed chromatin and to clarify its role in transcriptional activation using genetic, biochemical and pharmacological assays. We show that dMi-2 rapidly associates with activated HS loci, covering the entire transcribed region of the *hsp70* gene. dMi-2 recruitment is not affected when transcriptional elongation is blocked but is abrogated when PARP is inhibited. Indeed, we find that dMi-2 specifically binds PARP's oligomeric product PAR *in vitro.* Significantly, a dMi-2 mutant unable to bind PAR is not recruited to active HS loci *in vivo*. We have identified several regions of dMi-2 that bind PAR *in vitro*. These include the chromodomains and a series of K/R-rich motifs near the N-terminus. Further, dMi-2 depletion or expression of an inactive enzyme greatly decreases transcript levels, suggesting that dMi-2 actively supports efficient HS gene expression. Indeed, dMi-2 associates with nascent *hsp70* transcripts *in vivo* and ablation of dMi-2 function results in inefficient RNA processing. RNA and PAR compete for dMi-2 binding suggesting a two step process of dMi-2 association with HS genes: intial recruitment of dMi-2 is effected by its binding to PAR which is produced prior to the onset of transcription, dMi-2 then switches to interacting with the emerging nascent transcripts. Taken together, our results uncover PAR binding as a novel mechanism for the recruitment of the nucleosome remodeler dMi-2 to targeted sites of PARP activitation upon environmental stress and demonstrate that dMi-2 acts as a co-activator for the full transcriptional activation of HS genes. This study provides the first evidence for an *in vivo* function of PARylation in promoting the recruitment of a nucleosome remodeler to support the transcription of stress induced genes.

## Results

### dMi-2 is recruited to HS genes in a PARP–dependent manner

**Figure 1:**
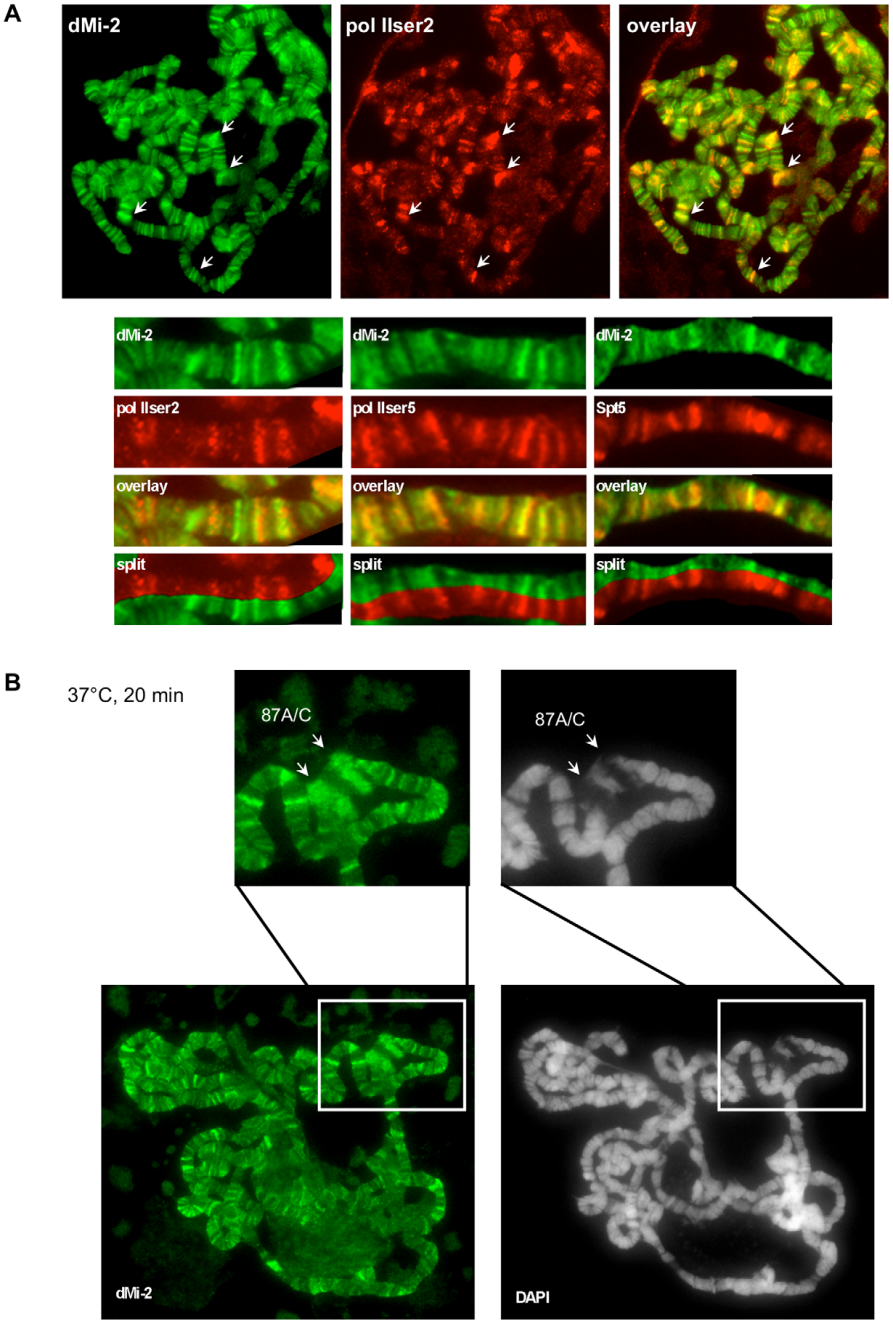
dMi-2 is recruited to HS genes. Immunofluorescence (IF) staining of polytene chromosomes with dMi-2, RNA polymerase II (pol IIser2 and pol IIser5), Spt5 antibodies and DAPI as indicated. (A) Untreated chromosomes. Arrows show prominent sites of colocalization. Lower panels show magnified sections of individual chromosome arms. (B) Heat shocked chromosomes. Upper panels show magnified section containing the *hsp70* loci 87A and 87C (arrows).

As shown previously, dMi-2 colocalised with active RNA polymerase II (Pol II) on polytene chromosomes [23] (Figure 1A). In addition, dMi-2 significantly colocalised with different forms of elongating Pol II (Ser2P and Ser5P) and elongation factors (Spt5). This suggests that dMi-2 may play an unanticipated role in active transcription. Upon HS, dMi-2 associated with the loci 87A and 87C which contain multiple copies of the *hsp70* gene (Figure 1B), further strengthening a potential link between dMi-2 and active transcription.

**Figure 2:**
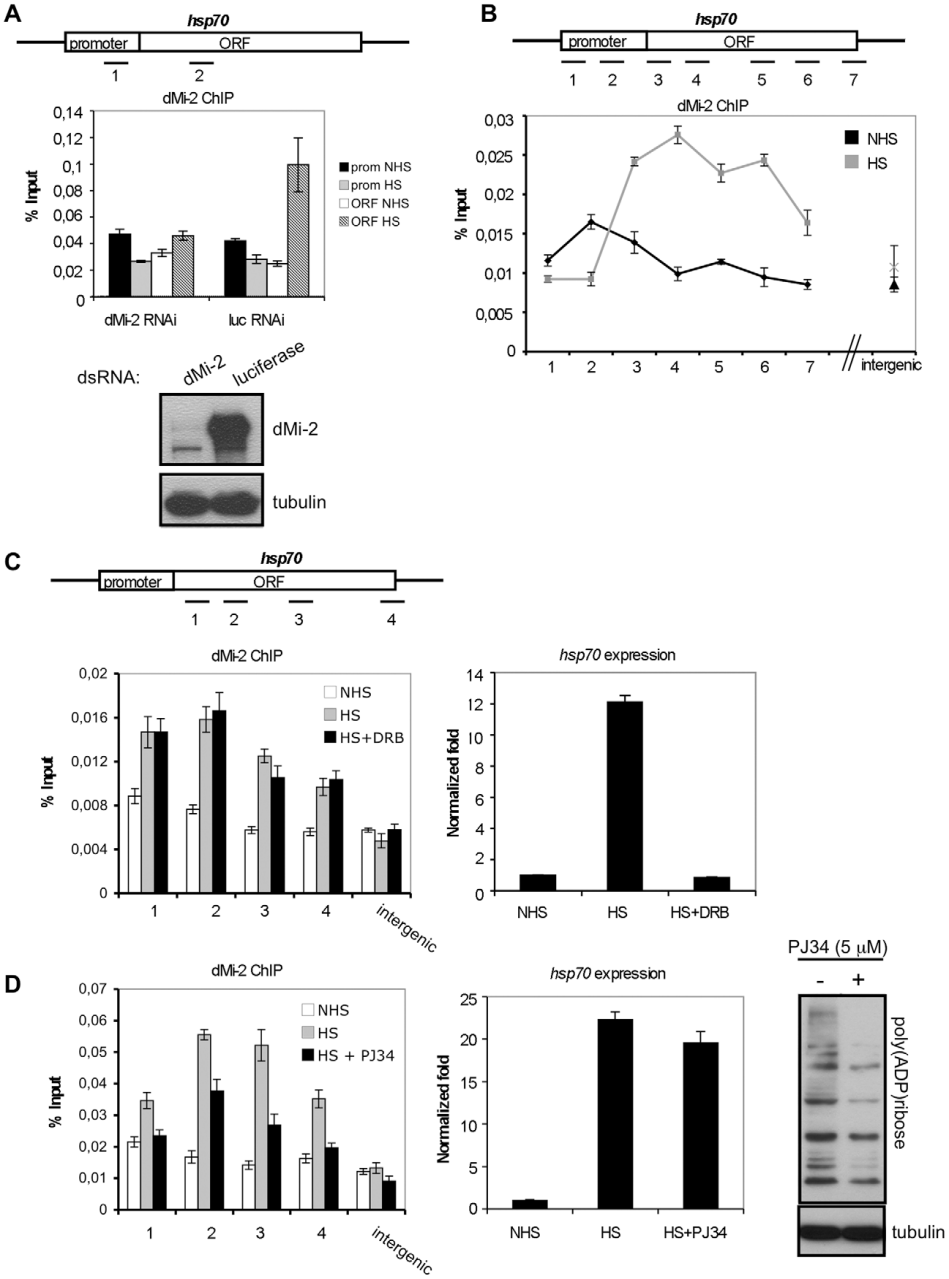
dMi-2 recruitment to HS genes requires PARP activity. (A and B) ChIP analyses of dMi-2 binding to the *hsp70* gene in Kc cells. (A) Upper panel: *hsp70* gene and position of amplimers analysed (1: centred at -154, 2: +681). Middle panel: dMi-2 ChIP from cells treated with dsRNA against luciferase or dMi-2 as indicated. prom (amplimer 1): promoter; ORF (amplimer 2): open reading frame; NHS: non heat shock; HS: heat shock. Lower panel: Verification of RNAi knockdown by Western blot. (B) Upper panel: *hsp70* gene and position of amplimers analysed (1: centred at -350; 2: -154; 3: +58; 4: +681; 5: +1702; 6: +2065; 7: +2549). Lower panel: dMi-2 ChIP from NHS (black graph) and HS (gray graph) cells. (C and D) Effect of elongation inhibitor DRB (C) and PARP inhibitor PJ34 (D) on dMi-2 recruitment to *hsp70* gene. *hsp70* gene and position of amplimers analysed are shown on top (1: centred at +58; 2: +681; 3: +1702; 4: +2549). Left panels: ChIP analyses of dMi-2 binding to *hsp70* gene. Right panels: RT-QPCR analysis of *hsp70* transcription. (D) Rightmost panel: anti-PAR Western blot of extracts from untreated and PJ34 treated Kc cells.

Chromatin immunoprecipitation (ChIP) analysis of dMi-2 binding to the activated *hsp70* gene in Kc cells revealed an enrichment of dMi-2 in the transcribed region (Figure 2A and 2B). dMi-2 association was detected as early as 2 min after HS and progressively increased for 20 min (Figure S1).

We considered three recruitment mechanisms:

First, dMi-2 might bind histone modifications enriched in actively transcribed genes, such as H3K4me3 or H3K36me3. However, we did not find a methylation sensitive interaction of recombinant dMi-2 with histone peptides in pulldown assays (data not shown).

Second, dMi-2 might bind and travel with RNA Pol II or elongation factors. This hypothesis predicts that HS-dependent dMi-2 recruitment to the transcribed part of *hsp70* is transcription-dependent. To test this hypothesis, we inhibited transcriptional elongation with DRB (Figure 2C). Although this treatment efficiently ablated production of *hsp70* transcripts, it did not significantly reduce HS-dependent recruitment of dMi-2. In addition, we failed to detect robust biochemical interactions of dMi-2 with RNA Pol II or elongation factors in co-immunoprecipitation assays (data not shown). We conclude that the HS-dependent recruitment of dMi-2 to the *hsp70* gene can be uncoupled from the transcriptional activity of *hsp70*.

Third, dMi-2 might be recruited by interaction with PAR, a modification that rapidly accumulates over the *hsp70* locus upon HS [3]. We therefore treated Kc cells with the small molecule PARP inhibitor PJ34 (Figure 2D). This led to a significant decrease of global PARylation levels, but did not abrogate *hsp70* transcription or nucleosome depletion (Figure 2D and Figure S2). Nevertheless, dMi-2 recruitment to *hsp70* was severely decreased during HS, suggesting that efficient PARylation of the locus is a requirement for stress-dependent enrichment of dMi-2.

### dMi-2 binds PAR

**Figure 3:**
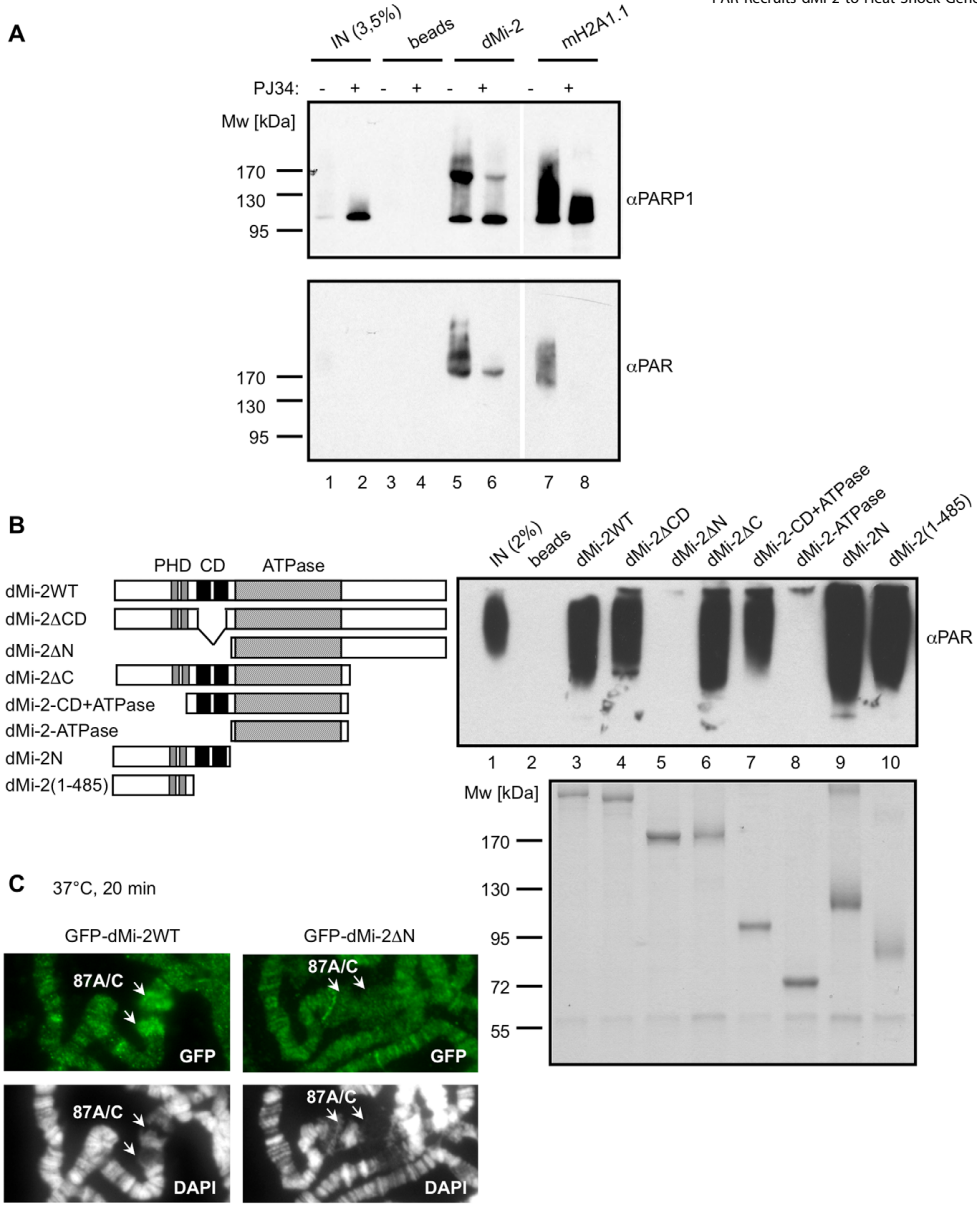
dMi-2 binds PAR. (A) PAR was synthesised *in vitro* by recombinant PARP1 in the presence (+) or absence (-) of PJ34. Reactions were incubated with control anti-Flag beads (beads) and beads loaded with dMi-2 or mH2a1.1 as indicated on top. Lanes 1, 2: input; Bound material was analysed by Western blot using PARP1 (upper panel) and PAR (lower panel) antibodies. (B) Mapping PAR binding regions. Left panel: Schematic representation of dMi-2 constructs used. Amino acid boundaries are as follows: dMi-2WT: dMi-2 1-1982; dMi-2ΔCD: dMi-2 Δ485-690; dMi-2ΔN: 691-1982; dMi-2ΔC: dMi-2 1-1271; dMi-2-CD+ATPase: dMi-2 484-1271; dMi-2-ATPase: dMi-2 691-1271; dMi-2N: dMi-2 1-690; dMi-2(1-485): dMi-2 1-485. Upper right panel: PAR binding assays with dMi-2 mutants were performed as in (A). dMi-2 mutants are shown on top. Bound material was analysed by anti-PAR Western blot. Lane 1: input. Lower right panel: Coomasie stained gel showing the dMi-2 constructs used. (C) Polytene chromosomes from transgenic larvae expressing GFP-dMi-2 transgenes (dMi-2WT and dMi-2ΔN) were analysed by IF using GFP antibody (green) and DAPI (gray). Arrows point to *hsp70* HS loci 87A and 87C.

To determine whether dMi-2 binds PAR directly, we auto-PARylated PARP1 *in vitro* and incubated the reaction with immobilised dMi-2. mH2A1.1 which contains a macrodomain known to interact with PAR was used as a positive control in this assay. Western blot analysis revealed that dMi-2, like mH2A1.1, bound PARylated PARP1 efficiently (Figure 3A). We confirmed that dMi-2 also interacted with radioactively labeled PARylated PARP1 (Figure S3). To ensure that dMi-2 interacted directly with the PAR polymer, we assayed binding to purified PAR using a dot blot assay (Figure S4). This verified the apparent direct interaction between dMi-2 and PAR.

Next, we sought to define the dMi-2 region required for PAR binding. We tested an array of dMi-2 truncation mutants for their ability to interact with PARylated PARP1 *in vitro* (Figure 3B). This revealed that the N-terminal region had a high affinity for PAR. Within this part of dMi-2, both the PHD finger containing region N-terminal of the chromodomains (aa 1-485) and (to a lesser extent) the chromodomains (aa 484-690) were capable of binding PAR. To verify these results we also tested binding of dMi-2 mutants to PAR in dot blot assays (Figure S4). We conclude that dMi-2 possesses at least two PAR-sensing regions that can function independently of each other.

### PAR–binding activity is required for dMi-2 recruitment to active HS loci

To assess the functional importance of dMi-2′s PAR binding activity, we compared recruitment of GFP-dMi-2 fusion proteins to the activated *hsp70* loci in transgenic flies (Figure 3C). GFP fused to full length dMi-2 and a GFP-dMi-2 fusion lacking the N-terminal PAR-binding regions were expressed to similar levels in 3rd instar larvae and correctly localised to salivary gland nuclei (Figure S5). Full length GFP-dMi-2 was enriched at active HS loci, the PAR binding mutant, however, failed to accumulate. This supports the notion that dMi-2 binding to PAR makes an important contribution to the recruitment of this nucleosome remodeler to the stress-activated *hsp70* gene.

### Mapping of the PAR–binding regions

The N-terminal PAR binding region of dMi-2 contains two highly conserved domains, a pair of PHD fingers (residues 377 to 484) and a tandem chromodomain (residues 488 to 673). We generated GST fusions containing these domains and tested their ability to bind PAR in dot blot assays (Figure S6). This confirmed that the chromomodomains can bind PAR independently. However, the PHD fingers did not display PAR binding activity.

**Figure 4:**
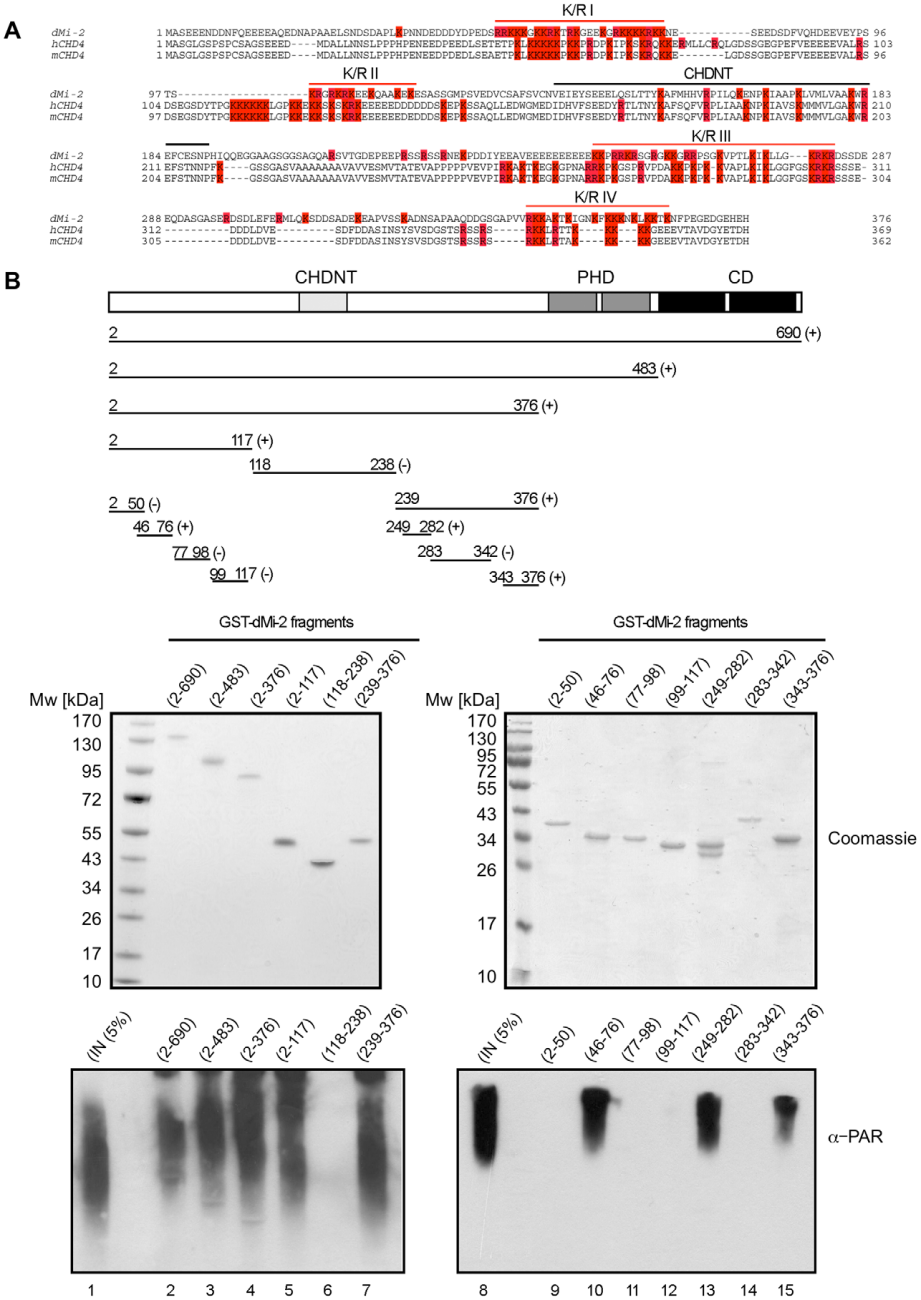
PAR–binding regions of dMi-2. (A) Multiple sequence alignment of N-terminus of dMi-2 and human and mouse CHD4. All K and R amino acid residues are coloured in red. Red lines indicate the four K/R rich regions. The black line indicates the CHDNT domain. (B) Mapping of PAR binding regions in the N-terminal part of dMi-2. Upper panel: Schematic representation of dMi-2 constructs used. Numbers indicate the amino acid borders of the constructs. (+) and (-) indicate binding to PAR. Middle panel: Coomasie stained gels with purified GST-dMi-2 fragments used for PARP pulldown assays. Lower panel: PAR binding assays with GST-dMi-2 fragments were performed as in Figure 3B. Bound material was analysed by anti-PAR Western blot. Lanes 1 and 8: inputs.

We next sought to better define the PAR binding region near the N-terminus of dMi-2. The N-terminal 375 residues of dMi-2 are characterised by a high content in charged residues (24% D/E, 21% R/K). This general feature is conserved between dMi-2 and mammalian CHD4 proteins (Figure 4A). In addition, these proteins share a region with high sequence similarity, the CHDNT domain (Pfam family PF08073). The function of this domain is not known. A number of diverse PAR binding motifs have recently been identified [24]–[26]. A common feature of these motifs is that they all contain several R/K residues that are interspersed by hydrophobic residues which often play critical roles in mediating PAR binding [24]–[26]. We subjected different dMi-2 fragments to the PAR binding assay, including four K/R-rich fragments (K/R I to IV in Figure 4A). This analysis revealed strong PAR binding activity for three of the four K/R-rich fragments (K/R I, K/R II and K/R IV; Figure 4B). By contrast, K/R-rich fragment II and a fragment encompassing the CHDNT domain failed to interact with PAR.

Taken together, our results suggest that dMi-2 contains multiple PAR binding regions in its N-terminus: three are characterised by a high content of basic amino acid residues (K/R I, K/R III and K/R IV) and one region containing the tandem chromodomain.

### dMi-2 interacts with nascent HS gene transcripts

**Figure 5:**
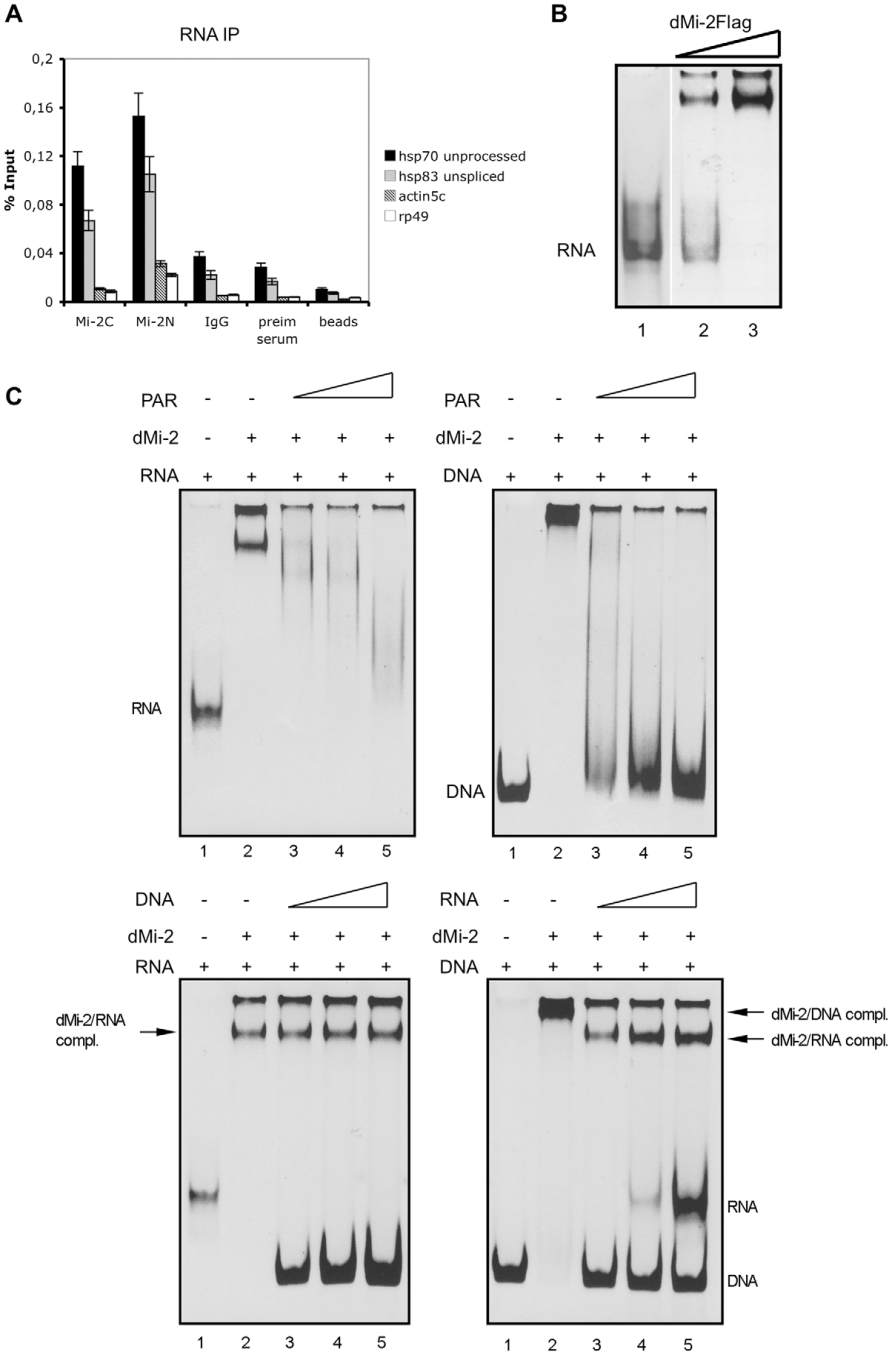
dMi-2 binds RNA. (A) RNA immunoprecipitation (RIP) of *hsp70* and *hsp83* unprocessed transcripts from heat shocked Kc cells. RIP was performed with two independent dMi-2 antibodies (dMi-2C and dMi-2N), IgG, preimmune serum and protein G beads as indicated. Primer pairs that specifically amplify *actin5c*, *rp49* and unprocessed *hsp70* and *hsp83* transcripts (see Figure 7) were used for RT-QPCR. (B) RNA electrophoretic mobility shift assay. Single stranded hsp70 RNA was incubated with recombinant dMi-2. Lane 2: 0.1 µg dMi-2, lane 3: 0.2 µg dMi-2, lane 1: no protein. RNA and RNA:protein complexes were resolved by electrophoresis and visualized with ethidium bromide. Position of unbound RNA probe is indicated. (C) Competition mobility shift assays. Upper left panel: Single stranded hsp70 RNA was incubated with 0.2 µg of recombinant dMi-2 in the absence or in the presence of increasing amounts of PAR polymer, as indicated. Upper right panel: hsp70 DNA was incubated with 0.2 µg of recombinant dMi-2 in the absence or in the presence of increasing amounts of PAR polymer, as indicated. The following mass ratios of RNA to PAR or DNA to PAR were used: lane 3 - 1∶1, lane 4 -1∶2, lane 5 -1∶4. Positions of unbound RNA and DNA probes are indicated. Lower left panel: Single stranded hsp70 RNA was incubated with 0.2 µg of recombinant dMi-2 in the absence or in the presence of increasing amounts of DNA, as indicated. Lower right panel: hsp70 DNA was incubated with 0.2 µg of recombinant dMi-2 in the absence or in the presence of increasing amounts of RNA, as indicated. The following weight ratios of RNA to DNA or DNA to RNA were used: line 3 - 1∶1, lane 4 -1∶2, lane 5 -1∶4. Positions of unbound RNA and DNA probes and dMi-2/DNA and dMi-2/RNA complexes are shown on the right.

PARylation of the *hsp70* locus has been proposed to assist in the opening of chromatin structure and to increase access of factors to DNA and nascent *hsp70* transcripts [1]. Given that dMi-2 localises to the entire transcribed region and given that PAR exhibits chemical and structural similarity to RNA, we speculated that dMi-2, once recruited, might interact with nascent *hsp70* RNA. We immunoprecipitated dMi-2 from nuclear extracts of heat shocked Kc cells and probed for the co-precipitation of nascent (unprocessed) hsp70 and hsp83 RNA (Figure 5A). Indeed, two independent dMi-2 antibodies precipitated these transcripts arguing for a physical, potentially direct interaction. In agreement with this, dMi-2 bound to single-stranded *hsp70* RNA in an electrophoretic mobility shift assay *in vitro* (Figure 5B).

Next, we performed competition assays to gain insight into the relative affinities of dMi-2 for DNA, RNA and PAR and to determine if dMi-2 can bind to several types of nucleic acid simultaneously or if binding is competitive. First, we tested dMi-2 binding to RNA and DNA, respectively, in the presence of increasing amounts of PAR in electrophoretic mobility shift assays (mass ratios 1∶1, 1∶2 and 1∶4; Figure 5C). In this assay, PAR was able to compete with RNA and DNA for dMi-2 binding. However, whereas dMi-2 no longer bound to DNA at a DNA:PAR mass ratio of 1∶2, residual dMi-2/RNA complexes were still detectable at an RNA:PAR mass ratio of 1∶4. This suggests that dMi-2 has a higher binding affinity for RNA than for DNA. We confirmed this hypothesis by incubating dMi-2 with different mass ratios of RNA and DNA (Figure 5C): At a DNA:RNA mass ratio of 1∶1, dMi-2/RNA complexes formed readily but dMi-2/DNA complexes were not detected. dMi-2/RNA complexes formed even at DNA:RNA mass ratios of 4∶1.

To test if RNA or DNA can compete with dMi-2 for binding to the branched PAR polymer we performed dot blot assays (Figure S7). RNA competed with immobilised PAR for binding to dMi-2 whereas DNA failed to do so.

Taken together, our results suggest that dMi-2 has a higher affinity for binding to RNA and PAR than for binding to DNA. In addition, dMi-2 appears to bind RNA and PAR in a mutually exclusive manner. These results are consistent with the hypothesis that dMi-2 is first recruited to HS loci by interaction with PAR (which is produced prior to and independent of transcription) and, once RNA synthesis has been strongly activated, switches to binding the nascent RNA.

### dMi-2 is required for efficient HS gene transcription and processing

**Figure 6:**
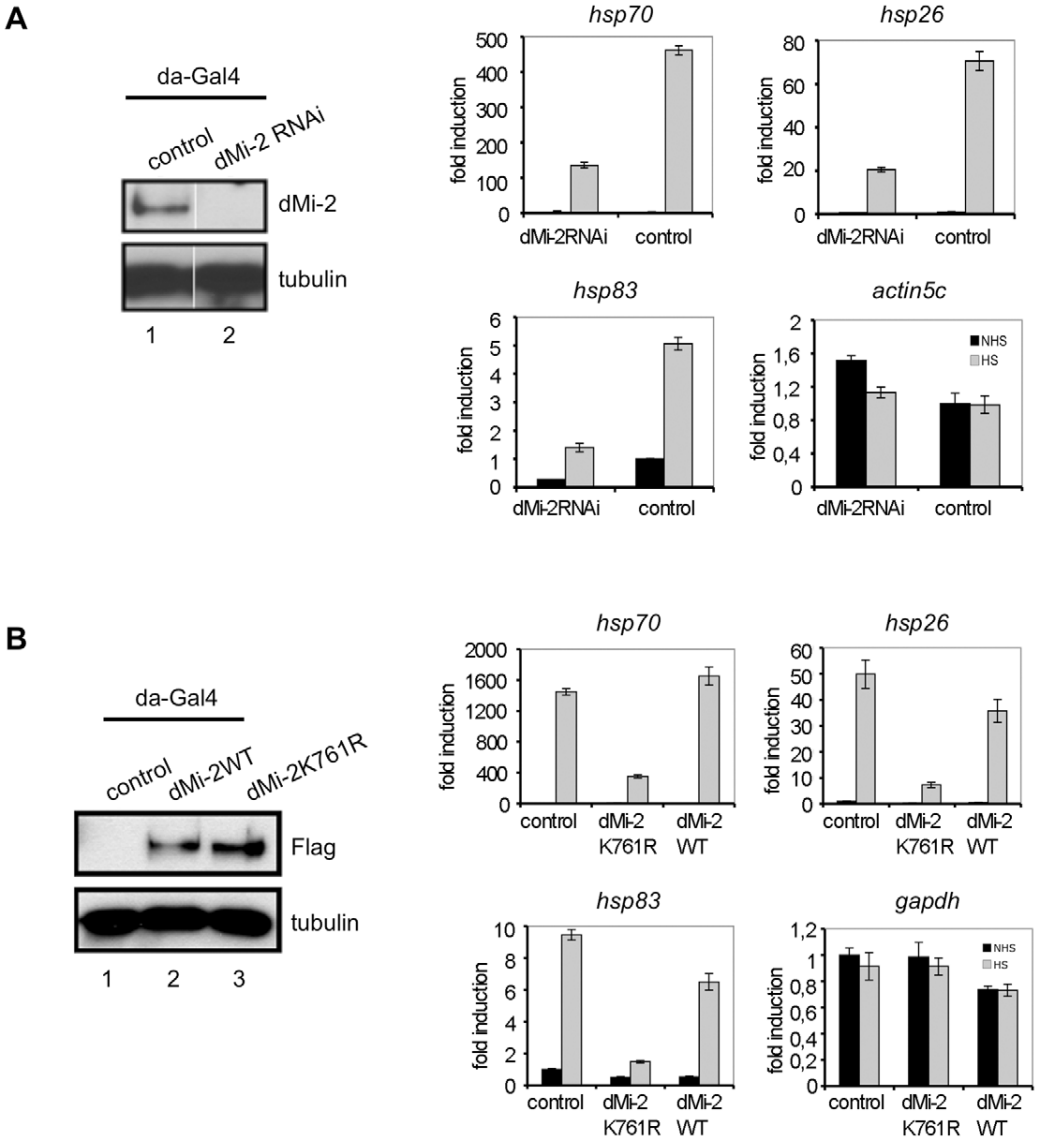
dMi-2 is required for efficient HS gene transcription. (A) Left panel: Verification of dMi-2 knockdown in control and dMi-2 RNAi larvae. Control flies and flies carrying an dMi-2 RNAi transgene under UAS control were crossed with a *da-GAL4* driver strain. Extracts from third instar larvae were subjected to Western Blot. Antibodies used are indicated on the right. Right panel: RT-QPCR analysis of *hsp70*, *hsp26*, *hsp83* and *actin5c* expression in control and dMi-2 RNAi larvae. Values are expressed relative to the value in NHS control larvae. (B) Left panel: Verification of dMi-2 transgene expression in larvae by anti-Flag Western blot. Right panel: RT-QPCR analysis of *hsp70*, *hsp26*, *hsp83* and *GAPDH* expression in control and transgenic larvae.

We hypothesised that dMi-2 binding to nascent RNA might influence *hsp70* transcription or processing. We used transgenic fly lines to deplete dMi-2 by RNAi (Figure 6A). We subjected transgenic larvae to HS and determined the HS gene transcription by RT-QPCR. Although *hsp70*, *hsp26* and *hsp83* genes were all activated by HS, transcript levels were severely reduced in dMi-2 depleted larvae compared to controls. Importantly, transcription of a housekeeping gene was not significantly affected. We conclude that dMi-2 makes a positive contribution to transcription and is essential for full HS gene activation in larvae.

We next determined whether dMi-2 enzymatic activity was required to activate HS genes. We generated transgenic fly lines overexpressing wild type dMi-2 or a dMi-2 mutant carrying a point mutation in the ATP binding site (K761R) predicted to prevent ATP binding (Figure 6B). Indeed, dMi-2K761R could not hydrolyse ATP *in vitro* (Figure S8). We subjected 3rd instar larvae to HS and determined effects on HS gene transcription as before. Whereas overexpression of wild type dMi-2 had little effect, levels of HS gene transcripts were greatly reduced in larvae overexpressing the enzymatically inactive dMi-2 (Figure 6B). We conclude that the ATPase activity of dMi-2 is essential for full HS gene activation.

**Figure 7:**
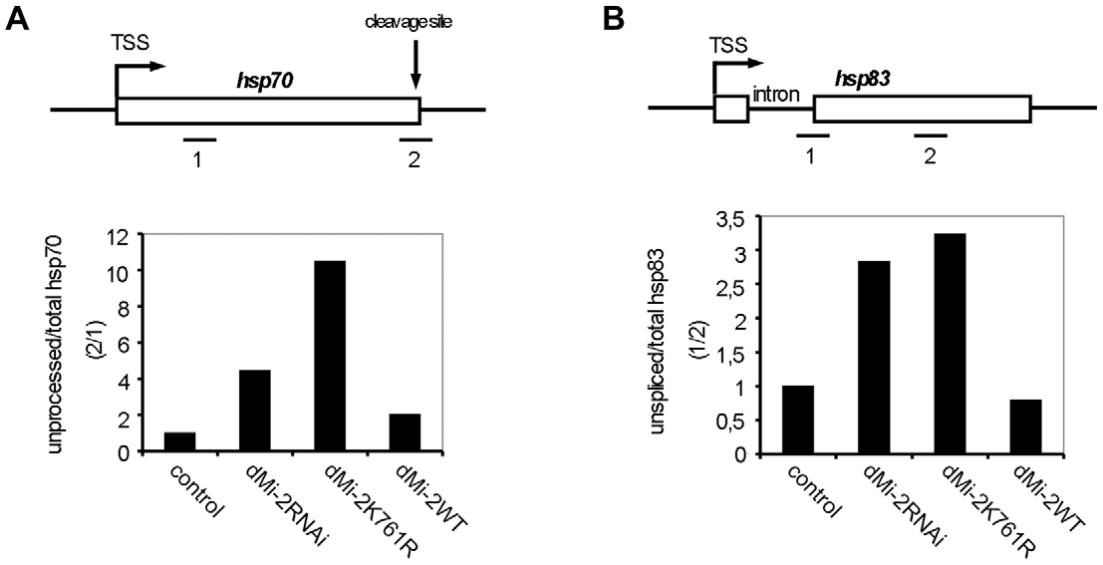
dMi-2 is required for efficient RNA processing. (A and B) Upper panels: Schematic representations of the *hsp70* and *hsp83* genes. RT-QPCR amplimers, *hsp70* cleavage site, *hsp83* intron and transcriptional start sites (TSS) are shown. (A) Lower panel: RT-QPCR from control and transgenic larvae. The ratio between 3′ unprocessed and total *hsp70* RNA was determined (*hsp70*: amplimer 2/amplimer 1). The ratio obtained for control larvae was set to 1, other ratios were expressed relative to this. (B) Lower panel: The ratio between unspliced and total *hsp83* RNA was determined (*hsp83:* amplimer 1/amplimer 2) and plotted as in (A).

Next, we sought to assess whether dMi-2 influences RNA processing. Because dMi-2 depletion and expression of enzymatically inactive dMi-2 resulted in an overall reduction of *hsp70* transcript levels we determined the ratio of 3′ unprocessed to total *hsp70* RNA as a measure of RNA processing efficiency. We reasoned that a mere reduction in *hsp70* activation (e.g. a reduction in the number of initiation events per time) would not change the ratio of unprocessed to total *hsp70* RNA. By contrast, processing defects might give rise to a higher relative proportion of unprocessed RNA and, therefore, to a higher unprocessed:total RNA ratio. Depletion of dMi-2 increased the relative proportion of unprocessed *hsp70* RNA (Figure 7A). An even more striking effect was observed in larvae overexpressing inactive dMi-2, whereas overexpression of wild type dMi-2 was of little consequence. Similar effects on 3′ RNA processing were observed with the *hsp83* gene (data not shown). *Hsp83* is one of the few HS genes possessing an intron. Therefore, we determined the ratio of unspliced to total *hsp83* transcripts in transgenic larvae (Figure 7B). Again, we observed a significant increase in the relative proportion of unspliced RNA in dMi-2-depleted larvae and in larvae overexpressing inactive enzyme. This suggests that dMi-2 activity is required for the efficient processing of HS gene transcripts and that dMi-2 affects both RNA 3′ end cleavage and splicing.

## Discussion

### dMi-2 associates with active genes

Mi-2 is strongly linked to transcriptional repression in both vertebrate and invertebrate organisms. Within NuRD and dMec complexes it contributes to the repression of cell type-specific genes [11], [14], [19]–[21]. Therefore, the widespread colocalisation of dMi-2 with active Pol II and elongation factors at many chromosomal sites is surprising and suggests that dMi-2 might play an unappreciated role during active transcription, at least (or specifically) during environmental stresses such as HS. Indeed, dMi-2 is recruited to HS genes within minutes of HS. This property is not shared by other chromatin remodelers: *Brahma* (BRM) is not enriched at HS puffs and HS gene activation is independent of BRM function ([27] and data not shown). Moreover, although *imitation switch* (ISWI) containing complexes are important for HS gene transcription, ISWI does not accumulate to high levels at active HS loci ([28], [29] and data not shown). Recruitment to HS puffs has previously been reported for *Drosophila* CHD1 [30]. Thus, accumulation at active HS genes is shared by at least two members of the CHD family of nucleosome remodelers but not by SWI/SNF and ISWI proteins.

### dMi-2 contributes to efficient HS gene transcription

Depletion of dMi-2 or a reduction of dMi-2 recruitment does not significantly perturb *hsp70* transcription in Kc cells and, therefore, dMi-2 is dispensable for HS gene activation in this system (Figure 2D and *data not shown*). By contrast, depletion of dMi-2 in larvae strongly decreases *hsp70*, *hsp26* and *hsp83* activation (Figure 6A). It is possible, that the RNAi-mediated depletion of dMi-2 is more efficient in transgenic flies compared to cell lines. In addition, it is believed that several factors contributing to HS gene activation are highly abundant or redundant in Kc cells but more limiting in other contexts. Accordingly, FACT and Spt6 are required for a HS gene activation in flies but are not essential in Kc cells [31], [32].

The strong decrease of HS gene activation in dMi-2 RNAi larvae indicates a positive contribution of dMi-2 to transcription *in vivo*. Overexpression of inactive dMi-2 also results in reduced HS gene transcription implying that its enzymatic activity is critical (Figure 6B). It is presently unclear whether this reflects a requirement for dMi-2 catalysed nucleosome remodeling or whether its activity is directed towards different substrates.

### dMi-2 contributes to efficient RNA processing

While dMi-2 could indirectly influence transcription by remodeling nucleosomes within the transcribed part of *hsp70*, its physical association with nascent HS gene transcripts argues for a more direct effect. Indeed, dMi-2 is not only required for high HS gene mRNA levels, but also affects the efficiency of co-transcriptional 3′ end formation and splicing. A role of chromatin remodelers in splicing has been suggested before: Both CHD1 and BRG1 bind components of the splicing apparatus [33], [34]. CHD1 associates with Pol II and binds nucleosomes containing H3K4me3, which are enriched near the 5′ end of active genes [34], [35]. BRG1 is present at the coding region of genes and influences splice site choice [33], [36]. It has been proposed that CHD1 and BRG1 physically recruit splicing factors but it is unclear if their ATPase activities play a role. Indeed, inactive BRG1 retains the ability to affect exon choice [33], [34]. Inefficient processing of the *hsp70* and *hsp83* transcripts is not only observed in larvae expressing reduced levels of dMi-2. Importantly, even stronger processing defects are generated by overexpression of inactive dMi-2 (Figure 7). This strongly suggests, for the first time, that the catalytic activity of a chromatin remodeler is required for correct co-transcriptional RNA processing. It remains to be determined whether dMi-2 nucleosome remodeling activity influences RNA processing indirectly, e.g. by altering Pol II elongation rates, or whether it has a more direct role.

### PAR–dependent recruitment of dMi-2

A series of complementary results support our hypothesis that dMi-2 interacts with PAR polymers that are rapidly synthesized at activated HS loci. First, the broad distribution of dMi-2 over the entire transcribed region correlates with the distribution of PAR polymer [3]. Second, pharmacological inhibition of PARP greatly decreases dMi-2 binding to activated *hsp70*. Third, dMi-2 directly binds PAR polymers *in vitro*. Fourth, an dMi-2 mutant unable to bind PAR also fails to localise to active HS loci. As discussed above, dMi-2 physically associates with nascent HS gene transcripts and binds RNA *in vitro*. While this interaction is potentially important for the efficiency of transcription and processing, it likely plays a minor role in dMi-2 targeting. Accordingly, inhibition of transcriptional elongation has no significant effect on dMi-2 recruitment (Figure 2C).

It is important to note, that while our results argue for an important role of PAR binding in the recruitment of dMi-2 to HS loci, we cannot exclude that protein-protein interactions with histone or non-histone proteins also play a role.

### dMi-2 PAR–binding

Our analysis indicates that dMi-2 harbours several PAR binding motifs in its N-terminal region. Polo and colleagues have recently demonstrated that human CHD4 is recruited to double stranded DNA breaks in a PARP-dependent manner [10]. They have mapped PAR binding activity to the region N-terminal of the ATPase domain of CHD4. This agrees well with our data and suggests that the PAR binding function of CHD4/dMi-2 has been conserved in evolution.

Two structural protein modules directly interact with PAR, the macrodomain and the PBZ domain; however, these domains are not present in dMi-2 [5], [7], [37]. In addition, several shorter PAR binding motifs have been identified [5], [26]. These motifs bear little sequence similarity but share the presence of several K/R residues which are interspersed by hydrophobic residues. Our results have uncovered three K/R-rich regions with PAR binding activity near the N-terminus of dMi-2. Two of these three K/R-rich regions (K/R III and K/R IV) consist of interspersed basic and hydrophobic residues and are therefore reminiscent of the previously described PAR binding motifs [24], [25], the third (K/R I) lacks hydrophobic residues completely. None of the three K/R regions matches the consensus PAR binding motifs. It is possible that a consensus motif should generally be chosen less stringently and that a high content of K and R-residues in these regions is sufficient to provide PAR binding activity in vitro. Further characterisation of these regions will be required to resolve this issue. In addition to the K/R regions, the tandem chromodomains of dMi-2 bind PAR *in vitro*. We have previously shown that the chromodomains are required for interacting with nucleosomal DNA *in vitro* [38]. Our new data suggests that these domains can interact with different nucleic acids.

### Different functions of PAR in HS gene transcription

Several potential molecular functions of PARylation at HS genes have been suggested. First, PARP activity is required for the rapid loss of nucleosomes at *hsp70* within the first two minutes after HS [1]. It has been suggested that PARylation of histones aids rapid nucleosome disassembly [1]. Second, at later stages of the HS response (20–60 minutes after HS), PARP activity is required to establish a compartment which restricts the diffusion of factors such as Pol II and Spt6 and promotes efficient factor recycling [4]. Our results suggest that PARylation carries out a third task, namely, to recruit factors via their direct interaction with PAR. The earliest time point when we can detect dMi-2 binding to *hsp70* is between 2 and 5 minutes after HS. This places dMi-2 recruitment between the early PARP-dependent nucleosome removal (0–2 minutes after HS) and effects of the transcription compartment (20–60 minutes after HS).

### PAR versus RNA binding

**Figure 8:**
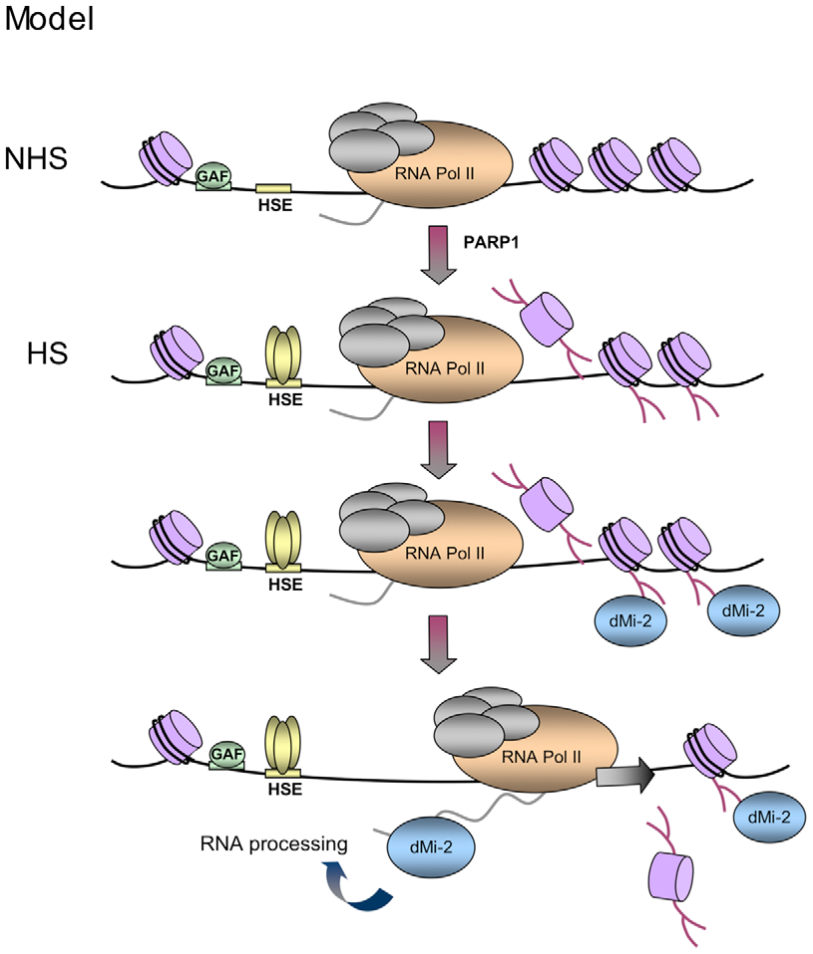
Model. Upon HS, PARylation of the locus creates binding sites for PAR-sensing regions of dMi-2. dMi-2 is recruited and, subsequently, interacts with nascent transcripts to support transcription and processing. GAF: GAGA Factor, HSE: HS elements.

The ability of dMi-2 to bind both PAR and RNA and the finding that RNA can compete for PAR binding to dMi-2 is consistent with the hypothesis that dMi-2 association with active HS genes is a two step process (Figure 8). We propose that dMi-2 is initially recruited via interaction with PAR polymers. Synthesis of these starts prior to the onset of *hsp70* transcription [1]. This results in a rapid local increase of the dMi-2 concentration. In the second step, when *hsp70* transcripts are produced by elongating RNA polymerase II at high rates, dMi-2 can switch from binding PAR to interacting with nascent transcripts.

### PAR signals rapid and efficient factor recruitment to chromatin during stress

Severe cellular stresses, such as DNA strand breaks and acute HS, must be dealt with quickly and efficiently. In both cases, a multitude of factors are rapidly recruited to orchestrate the repair of DNA and the massive transcriptional activation of HS genes, respectively. We postulate that rapid synthesis of PAR polymers at both DNA damage sites and HS genes affords an efficient mechanism to recruit chromatin remodelers and other factors. It has recently been shown that PARylation of DNA breaks is instrumental in recruiting chromatin remodelers, including mammalian dMi-2 homologs, to damaged sites [8], [9], [10], [39], [40], [41]. Here, we show that dMi-2′s recruitment to activated HS genes requires PARP activity and that dMi-2 binds PAR directly. The high local concentration of PAR polymers at DNA breaks and HS genes might exploit the general affinity of dMi-2 for nucleic acids. Indeed, dMi-2 binds both DNA and RNA as well as PAR *in vitro* ([38] and this study). In this manner, PAR polymers might act as a scaffold to redirect dMi-2 to chromatin regions where high levels of dMi-2 activity are required, thus acting as a stress-dependent, transient affinity site for chromatin remodeling and possibly RNA processing activities (Figure 8). Our results highlight a signaling and scaffolding function for PARP activity during transient environmental stresses other than DNA damage, suggesting that PARylation carries out important modulatory functions in the stress-dependent reprogramming of nuclear activities.

## Materials and Methods

### Chromatin immunoprecipitation (ChIP)

Kc cell HS treatment and ChIP was performed as decribed using dMi-2C antibody [14], [42]. For primer sequences see Dataset S2. Triplicate mean values of percentage input DNA and standard deviations are plotted. dMi-2 knockdown by RNAi was described previously [23]. For RNAi primer sequences see Dataset S4.

### Pharmacological treatments

Kc cells were treated with 125 µM DRB (Sigma) to inhibit transcription and with 5 µM PJ34 (Alexis) to inhibit PARP activity for 20 min before subjecting cells to HS.

### Polytene chromosomes

Chromosomes were prepared as before [23]. The following antibodies were used: Primary antibodies: anti-dMi-2N (rabbit) 1∶200, anti-pol II (mouse H5, Covance) 1∶50, anti-GFP (rabbit, Abcam) 1∶50, anti-Spt5 (guinea pig) 1∶200. Secondary antibodies: Alexa Fluor 488 goat anti-rabbit 1∶200, Alexa Fluor 546 goat anti-mouse or anti-guinea pig 1∶200 (Invitrogen).

Analysis was performed with a Zeiss fluorescence microscope (Axioplan).

### Generation of baculovirus and GST-fusion vectors

For baculovirus production, dMi-2 mutants (aa 1-691) and (aa 1-485) were generated by PCR using appropriate sets of primers and cloned with NotI and XbaI into the pVL1392 transfer vector. Vectors for dMi-2 WT and other mutants were described previously [38]. dMi-2 GST-fusion fragments were generated by PCR using appropriate sets of primers and cloned with NotI and SalI into the pGEX4T1 vector. All constructs were verified by DNA sequencing. For primer sequences see Dataset S1.

### Preparation of protein extracts, purification of recombinant proteins, and ATPase assay

Protein extracts from 3rd instar larvae were prepared as described in [14].

Purification of recombinant dMi-2 and ATPase assays are described [23]. Recombinant mH2A1.1 was purified as in [43]. GST-fusion proteins were expressed in *E.coli* BL21(DE3) and purified with Glutathione Sepharose 4 Fast flow (GE Healthcare) according to the manufacturer's instructions.

### Electrophoretic mobility shift assays (EMSA)

A typical DNA or RNA binding reaction (25 µl) was performed in the presence of 0.2 µg of dMi-2F and 80 ng of nucleic acid (DNA or ssRNA) in 40 mM KCl, 20 mM Tris pH 7.6, 1.5 mM MgCl_2_, 0.5 mM EGTA, 10% glycerol, BSA (200 ng/µl), 1 mM DTT (supplemented with 0.4 units of RNAsin). For competition assays, samples were preincubated for 15 min at 26°C before the different amounts of competitor (PAR or DNA or RNA) were added. Reactions were further incubated at 26°C for 75 min. Products were analyzed on 6% native PAA gel and visualized with ethidium bromide (EtBr) staining. ssRNA was synthesized by in vitro transcription using a fragment of hsp70 DNA as a template. This template (also used for the DNA bandshift assays) was produced by PCR amplification of cDNA derived from heat shocked Kc cells using the following primers: T7-hsp70_f - TAATACGACTCACTATAGGGCCTACGGACTGGACAAGAAC and hsp70_r -AGGGTTGGAGCGCAGATCCTTCTTGTAC.

### RNA isolation and RT-QPCR

Total RNA was isolated from 3rd instar larvae using PeqGold total RNA Kit (PeqLab). 10-12 larvae from each cross were pestled in 400 µl of lysis buffer before loading the material on the column. 1 µg of RNA was reverse transcribed by incubation with 0.3 µg of random primers (Invitrogene) and 100 U of M-MLV reverse transcriptase (Invitrogen). cDNA synthesis was performed according to the manufacturer's protocol. cDNA was analyzed by QPCR using Absolute SybrGreen Mix (Thermo Fisher) and the Mx3000P real-time detection system (Agilent). For primer sequences used in RT-QPCR see Dataset S3. All amplifications were performed in triplicate using 0.6 µl of cDNA per reaction. Triplicate mean values were calculated according to the ΔΔCt quantification method using *rp49* gene transcription as reference for normalization. Relative mRNA levels in uninduced control larvae were set to 1 and other values were expressed relative to this. The RT-QPCR results were reproduced several times using independent fly crosses and representative data sets are shown.

### RNA immunoprecipitation

RNA immunoprecipitation was performed as described previously [44]. Briefly, Kc cells were crosslinked as for ChIP. Cells were washed once with PBS buffer and lysed on ice for 15 min in FA buffer (50 mM Hepes- KOH, pH 7,6, 140 mM NaCl, 1% Triton X-100, 0,1% sodium deoxycholate, proteinase inhibitors, RNAsin (100 u/ml of buffer)). Cells were sonicated, spun down and chromatin was digested with DNAse I. The chromatin containing solution was adjusted to 25 mM MgCl_2_ and 5 mM CaCl_2_. 1 ul of DNAse I (Qiagen) was added and reactions were incubated for 10 min at room temperature and then stopped with 20 mM EDTA. Chromatin was spun down for 10 min (13000 rpm) at 4°C. 300 µl of chromatin was used for IP. 2 µl of anti-dMi-2(C) and anti-dMi-2(N) antibodies, 2 µl rabbit IgG, 2 µl rabbit preimmuneserum and beads only (control) and were used for IP. Samples were incubated over night at 4°C. RNA-protein complexes were precipitated with 30 ul of 50% protein G Sepharose beads for 2 hr at 4°C. IPs were washed 5 times in FA buffer, twice with TE buffer and eluted twice with 100 µl of elution buffer (100 mm Tris HCl, pH 8,0, 10 mM EDTA, 1% SDS) – once at room temperature and once at 65°C. All buffers were supplemented with RNAse inhibitor (RNAsin, Promega). All samples were digested with proteinase K for 1 hr at 42°C and decrosslinking was performed at 65°C over night. Immunoprecipitated RNA was purified using PeqGold total RNA Kit (PeqLab), digested with DNAse on the column and eluted with 30 µl of RNAse free dH20. cDNA was synthesized with 10 µl of eluted RNA and 2 µl of input with random hexamers and analysed by Q-PCR with appropriate primer pairs.

### PARP/PAR pulldowns

Non-radioactive PAR synthesis was performed according to the standard protocol [45]. Briefly, PARP reactions were set up in a final volume of 0.5 ml: 2 µg recombinant Parp1, 100 mM Tris-HCl, pH 7.5, 50 mM NaCl, 10 mM MgCl2, 2 µg/ml DNA oligonucleotides, 1 mM NAD+, 1 mM DTT. Reactions were incubated at 37°C for 25 min. PJ34 inhibitor was added before the reaction to control samples to a final concentration of 5 µM. All reactions were stopped with PJ34. Control beads and beads with bound proteins (dMi-2, dMi-2 mutants and mH2A1.1 or GST fusions) were equilibrated in binding buffer (50 mM Tris, pH 8.0, 0,2 mM DTT, 4 mM MgCl2, 200 mM NaCl, 0,1% NP-40). 10 µl of bead-bound proteins were used for each pulldown. Pulldowns were performed with the whole PARP reaction (0.5 ml) and 500 µl of binding buffer (for baculovirus expressed proteins) or in 250 µl of PARP reaction and 250 µl of binding buffer (for GST fusions) for 1 hr at 4°C. After extensive washing (5 times), beads were boiled in SDS loading buffer, loaded on 4–12% gradient SDS-Page gels and analysed by Western blot. For Western blot anti-PAR (10H, 1∶500) antibodies were used. mH2A1.1 was used as a positive control. Radioactive pulldown reactions were prepared in the same way, in the presence of 2 µl of radioactive NAD+ (PerkinElmer). After washing, samples were resuspended in 30 µl of SDS-loading buffer and 10 µl was resolved by SDS PAGE. The gel was dried and and subjected to autoradiography.

Generation of transgenic fly strains and the PAR dot blot assay are described in Text S1.

## Supporting Information

Dataset S1 Sequences of primers used for cloning.
(DOCX)

Dataset S2 Sequences of primers used for ChIP.
(DOCX)

Dataset S3 Sequences of primers used for RT-QPCR.
(DOCX)

Dataset S4 Sequences of primers used for ds RNA synthesis (RNAi).
(DOCX)

Figure S1 Kinetic analysis of dMi-2 binding to hsp70 gene during heat shock. dMi-2 binding to hsp70 gene was determined by ChIP under NHS conditions and at different time points following heat shock as indicated. Amplimers were centered as follows: 1, -154 bp; 2, +58 bp; 3, +681 bp; 4, +1427 bp; 5, + 2549 bp.
(TIF)

Figure S2 H3 ChIP on *hsp70* gene upon PJ34 treatment. dMi-2 binding to hsp70 gene was determined by ChIP under NHS conditions and at different time points in the absence or in the presence of PJ34, as indicated. Amplimers were centered as follows: 1, -154 bp; 2, +58 bp; 3, +681 bp; 4, +1427 bp; 5, + 2549 bp.
(TIF)

Figure S3 Pulldown with whole PAR reaction. Experiment was performed as in Figure 3A with a difference that radioactive NAD+ was used for PAR synthesis. Samples were run on the gel, gel was dried and exposed overnight on the X-ray film.
(TIF)

Figure S4 PAR binding assay. Dot blot with purified PAR. BSA or recombinant dMi-2 WT and indicated mutants were spotted on the nitrocellulose and incubated with PAR. Upon extensive washes, membrane was subjected to Western Blot analysis with anti-PAR antibodies. After stripping, membrane was probed with anti-Flag antibodies to monitor the amount of proteins spotted.
(TIF)

Figure S5 PAR binding assay. Upper panel: Dot blot with purified PAR. GST-fusion proteins and GST were spotted on the nitrocellulose and incubated with PAR. Upon extensive washes with low salt (150 mM) or high salt (500 mM), membranes were subjected to Western Blot analysis with anti-PAR antibodies. Lower panel: Coomasie stained gel with purified proteins used for PAR binding assay. Chromo - chromodomains of dMi-2 (aa 488-712), PHDs – PHD fingers of dMi-2 (aa 377-490).
(TIF)

Figure S6 Expression analysis of GFP-tagged transgenes. Left panel – whole salivary glands from flies crossed to the salivary gland-specific sgs58ABGAL4 driver were analysed for GFP expression. Right panel: larval extracts derived from control (w1118) larvae (line 1) and larvae expressing GFPtagged dMi-2WT (lane 3) or dMi-2ΔN transgene (lane 2) crossed to *daughterless*-GAL4 driver were analysed by western blot using GFP antibodies.
(TIF)

Figure S7 Competition of PAR binding with RNA and DNA. (A) Dot blot with purified PAR. dMi-2 WT was spotted on the nitrocellulose and incubated with PAR. Upon extensive washes membranes were subjected to Western Blot analysis with anti-PAR antibodies (upper panel), Ponceau staining indicates the amount of protein spotted (lower panel). When indicated, membranes were preincubated with increasing amounts of RNA (lanes: 2,3 and 4) followed by incubation with PAR. Lane 1: dMi-2 was preincubated with buffer only. (A) Dot blot with purified PAR. dMi-2 WT was spotted on the nitrocellulose and incubated with PAR. When indicated, membranes were preincubated with increasing amounts of DNA (lanes: 2,3 and 4) followed by incubation with PAR. Lane 1: dMi-2 was preincubated with buffer only.
(TIF)

Figure S8 dMi-2 K761R mutant is catalytically inactive. Upper panel: a Coomasie gel with dMi-2 WT and dMi-2 K761R mutant. Lower panel: ATPase assay with wild type and mutant form of dMi-2 in the presence of nucleosomes.
(TIF)

Text S1 Supporting protocols: Fly strains, transgenesis and dot blot assay.
(DOC)

## References

[1] Petesch SJ, Lis JT (2008). Rapid, transcription-independent loss of nucleosomes over a large chromatin domain at Hsp70 loci.. Cell.

[2] Winegarden NA, Wong KS, Sopta M, Westwood JT (1996). Sodium salicylate decreases intracellular ATP, induces both heat shock factor binding and chromosomal puffing, but does not induce hsp 70 gene transcription in Drosophila.. J Biol Chem.

[3] Tulin A, Spradling A (2003). Chromatin loosening by poly(ADP)-ribose polymerase (PARP) at Drosophila puff loci.. Science.

[4] Zobeck KL, Buckley MS, Zipfel WR, Lis JT (2010). Recruitment Timing and Dynamics of Transcription Factors at the Hsp70 Loci in Living Cells.. Molecular Cell.

[5] Krishnakumar R, Kraus WL (2010). The PARP side of the nucleus: molecular actions, physiological outcomes, and clinical targets.. Mol Cell.

[6] Till S, Ladurner AG (2009). Sensing NAD metabolites through macro domains.. Front Biosci.

[7] Timinszky G, Till S, Hassa PO, Hothorn M, Kustatscher G (2009). A macrodomain-containing histone rearranges chromatin upon sensing PARP1 activation.. Nat Struct Mol Biol.

[8] Ahel D, Horejsi Z, Wiechens N, Polo SE, Garcia-Wilson E (2009). Poly(ADP-ribose)-dependent regulation of DNA repair by the chromatin remodeling enzyme ALC1.. Science.

[9] Gottschalk AJ, Timinszky G, Kong SE, Jin J, Cai Y (2009). Poly(ADP-ribosyl)ation directs recruitment and activation of an ATP-dependent chromatin remodeler.. Proc Natl Acad Sci U S A.

[10] Polo SE, Kaidi A, Baskcomb L, Galanty Y, Jackson SP (2010). Regulation of DNA-damage responses and cell-cycle progression by the chromatin remodelling factor CHD4.. Embo J.

[11] Fujita N, Jaye DL, Geigerman C, Akyildiz A, Mooney MR (2004). MTA3 and the Mi-2/NuRD complex regulate cell fate during B lymphocyte differentiation.. Cell.

[12] Murawsky CM, Brehm A, Badenhorst P, Lowe N, Becker PB (2001). Tramtrack69 interacts with the dMi-2 subunit of the Drosophila NuRD chromatin remodelling complex.. EMBO Rep.

[13] Marfella CG, Imbalzano AN (2007). The Chd family of chromatin remodelers.. Mutat Res.

[14] Kunert N, Wagner E, Murawska M, Klinker H, Kremmer E (2009). dMec: a novel Mi-2 chromatin remodelling complex involved in transcriptional repression.. Embo J.

[15] Feng Q, Zhang Y (2001). The MeCP1 complex represses transcription through preferential binding, remodeling, and deacetylating methylated nucleosomes.. Genes Dev.

[16] Le Guezennec X, Vermeulen M, Brinkman AB, Hoeijmakers WA, Cohen A (2006). MBD2/NuRD and MBD3/NuRD, two distinct complexes with different biochemical and functional properties.. Mol Cell Biol.

[17] Lyko F, Beisel C, Marhold J, Paro R (2006). Epigenetic regulation in Drosophila.. Curr Top Microbiol Immunol.

[18] Kehle J, Beuchle D, Treuheit S, Christen B, Kennison JA (1998). dMi-2, a hunchback-interacting protein that functions in polycomb repression.. Science.

[19] Kim J, Sif S, Jones B, Jackson A, Koipally J (1999). Ikaros DNA-binding proteins direct formation of chromatin remodeling complexes in lymphocytes.. Immunity.

[20] Koipally J, Renold A, Kim J, Georgopoulos K (1999). Repression by Ikaros and Aiolos is mediated through histone deacetylase complexes.. Embo J.

[21] Reddy BA, Bajpe PK, Bassett A, Moshkin YM, Kozhevnikova E (2010). Drosophila transcription factor Tramtrack69 binds MEP1 to recruit the chromatin remodeler NuRD.. Mol Cell Biol.

[22] Stielow B, Sapetschnig A, Kruger I, Kunert N, Brehm A (2008). Identification of SUMO-dependent chromatin-associated transcriptional repression components by a genome-wide RNAi screen.. Mol Cell.

[23] Murawska M, Kunert N, van Vugt J, Langst G, Kremmer E (2008). dCHD3, a novel ATP-dependent chromatin remodeler associated with sites of active transcription.. Mol Cell Biol.

[24] Gagne JP, Hunter JM, Labrecque B, Chabot B, Poirier GG (2003). A proteomic approach to the identification of heterogeneous nuclear ribonucleoproteins as a new family of poly(ADP-ribose)-binding proteins.. Biochem J.

[25] Pleschke JM, Kleczkowska HE, Strohm M, Althaus FR (2000). Poly(ADP-ribose) binds to specific domains in DNA damage checkpoint proteins.. J Biol Chem.

[26] Zhang Y, Liu S, Mickanin C, Feng Y, Charlat O RNF146 is a poly(ADP-ribose)-directed E3 ligase that regulates axin degradation and Wnt signalling.. Nat Cell Biol.

[27] Armstrong JA, Papoulas O, Daubresse G, Sperling AS, Lis JT (2002). The Drosophila BRM complex facilitates global transcription by RNA polymerase II.. Embo J.

[28] Badenhorst P, Xiao H, Cherbas L, Kwon SY, Voas M (2005). The Drosophila nucleosome remodeling factor NURF is required for Ecdysteroid signaling and metamorphosis.. Genes Dev.

[29] Deuring R, Fanti L, Armstrong JA, Sarte M, Papoulas O (2000). The ISWI chromatin-remodeling protein is required for gene expression and the maintenance of higher order chromatin structure in vivo.. Mol Cell.

[30] Kelley DE, Stokes DG, Perry RP (1999). CHD1 interacts with SSRP1 and depends on both its chromodomain and its ATPase/helicase-like domain for proper association with chromatin.. Chromosoma.

[31] Ardehali MB, Yao J, Adelman K, Fuda NJ, Petesch SJ (2009). Spt6 enhances the elongation rate of RNA polymerase II in vivo.. Embo J.

[32] Saunders A, Werner J, Andrulis ED, Nakayama T, Hirose S (2003). Tracking FACT and the RNA polymerase II elongation complex through chromatin in vivo.. Science.

[33] Batsche E, Yaniv M, Muchardt C (2006). The human SWI/SNF subunit Brm is a regulator of alternative splicing.. Nat Struct Mol Biol.

[34] Sims RJ, Millhouse S, Chen CF, Lewis BA, Erdjument-Bromage H (2007). Recognition of trimethylated histone H3 lysine 4 facilitates the recruitment of transcription postinitiation factors and pre-mRNA splicing.. Mol Cell.

[35] Srinivasan S, Armstrong JA, Deuring R, Dahlsveen IK, McNeill H (2005). The Drosophila trithorax group protein Kismet facilitates an early step in transcriptional elongation by RNA Polymerase II.. Development.

[36] Tyagi A, Ryme J, Brodin D, Ostlund Farrants AK, Visa N (2009). SWI/SNF associates with nascent pre-mRNPs and regulates alternative pre-mRNA processing.. PLoS Genet.

[37] Ahel I, Ahel D, Matsusaka T, Clark AJ, Pines J (2008). Poly(ADP-ribose)-binding zinc finger motifs in DNA repair/checkpoint proteins.. Nature.

[38] Bouazoune K, Mitterweger A, Langst G, Imhof A, Akhtar A (2002). The dMi-2 chromodomains are DNA binding modules important for ATP-dependent nucleosome mobilization.. Embo J.

[39] Chou DM, Adamson B, Dephoure NE, Tan X, Nottke AC (2010). A chromatin localization screen reveals poly (ADP ribose)-regulated recruitment of the repressive polycomb and NuRD complexes to sites of DNA damage.. Proc Natl Acad Sci U S A.

[40] Larsen DH, Poinsignon C, Gudjonsson T, Dinant C, Payne MR (2010). The chromatin-remodeling factor CHD4 coordinates signaling and repair after DNA damage.. J Cell Biol.

[41] Smeenk G, Wiegant WW, Vrolijk H, Solari AP, Pastink A (2010). The NuRD chromatin-remodeling complex regulates signaling and repair of DNA damage.. J Cell Biol.

[42] Boehm AK, Saunders A, Werner J, Lis JT (2003). Transcription factor and polymerase recruitment, modification, and movement on dhsp70 in vivo in the minutes following heat shock.. Mol Cell Biol.

[43] Kustatscher G, Hothorn M, Pugieux C, Scheffzek K, Ladurner AG (2005). Splicing regulates NAD metabolite binding to histone macroH2A.. Nat Struct Mol Biol.

[44] Gilbert C, Svejstrup JQ (2006). RNA immunoprecipitation for determining RNA-protein associations in vivo.. Curr Protoc Mol Biol Chapter.

[45] Karras GI, Kustatscher G, Buhecha HR, Allen MD, Pugieux C (2005). The macro domain is an ADP-ribose binding module.. Embo J.

