# Supplementary material for: Stress-Induced PARP Activation Mediates Recruitment of Drosophila Mi-2 to Promote Heat Shock Gene Expression
Source: PLoS Genet. 2011 Jul 28;7(7):e1002206. doi: 10.1371/journal.pgen.1002206 (PMC3145624; doi:10.1371/journal.pgen.1002206)
Supplement: Dataset S1 — Sequences of primers used for cloning. (DOCX) [file pgen.1002206.s001.docx]

Primers used for cloning:

| Primer name | Sequence |
| --- | --- |
| dMi-2for | 5´AAAGCGGCCGCATGGCATCGGAGGAAGAG3´ |
| dMi-2(1-691)rev | 5´CCGTCTAGACTACTTGTCATCGTCGTCCTTGTAGTCGCAGCTGCAACGAGGACAG3´ |
| dMi-2(1-485)rev | 5´CCGTCTAGACTACTTGTCATCGTCGTCCTTGTAGTCGACCTTGAGCTTGGACTTG3´ |
| eGFP_for | 5´AGCGAATTCATGGCACCAAAGAAGAAG3´ |
| eGFP_rev | 5´ATTCTCGAGCTAGCGGCCGCTGAGTCCGGACTTGTACAG3´ |
| eGFPdMi-2WT_for | 5´ATAGCGGCCGCGCATCGGAGGAAGAGAATG3´ |
| eGFPdMi2WT_rev | 5´AGGTCTAGACTAGACGCCGGAATTATTCG3´ |
| eGFPdMi-2ΔN_for | 5´ATAGCGGCCGCGAGGACGACGAGGATCG3´ |
| dMi-2(488-712)for | 5´TGTGAATTCGACTCATGTCCCTCCGCCTATC3´ |
| dMi-2(488-712)rev | 5´ATAGCGGCCGCCTAATACTTCTTCTTCAGATC3´ |
| dMi-2(377-490)for | 5´GCGCCATGGCACAGGACTACTGCGAGGTG3´ |
| dMi-2(377-490)rev | 5´ACAGCGGCCGCTCAAGCCTTGCCAGTGAG3´ |
| dMi-2(2-690)for | 5´ATAgtcgacttGCATCGGAGGAAGAGAATG3´ |
| dMi-2 (2-690)rev | 5´ATAgcggccgcctaGACCTTGAGCTTGGACTTGC3´ |
| dMi-2 (2-483)rev | 5´ATAgcggccgcctaGCAGCTGCAACGAGGACAG3´ |
| dMi-2 (2-376)rev | 5´ATAgcggccgcctaGTGCTCATGCTCGCCATC3´ |
| dMi-2 (2-117)for | 5´ATAgcggccgcctaTGCCGACTCCTTCTCCTTG3´ |
| dMi-2 (118-238)for | 5´ATAgtcgacttTCATCCGGAATGCCATCTG3´ |
| dMi-2 (118-238)rev | 5´ATAgcggccgcctaGACGGCCTCCTCGTAAATG3´ |
| dMi-2 (239-376)for | 5´ATAgcggccgcctaGACGGCCTCCTCGTAAATG3´ |
