# Supplementary material for: Stress-Induced PARP Activation Mediates Recruitment of Drosophila Mi-2 to Promote Heat Shock Gene Expression
Source: PLoS Genet. 2011 Jul 28;7(7):e1002206. doi: 10.1371/journal.pgen.1002206 (PMC3145624; doi:10.1371/journal.pgen.1002206)
Supplement: Dataset S2 — Sequences of primers used for ChIP. (DOCX) [file pgen.1002206.s002.docx]

ChIP pimers:

| Primer name | Sequence (from 5´ to 3´) |
| --- | --- |
| hsp70-350_f | TGGGTGTCTACCAACATGGCAA |
| hsp70-350_r | ATGAGGCGTTCCGAATCTGTGA |
| hsp70-154_f | TGCCAGAAAGAAAACTCGAGAAA |
| hsp70-154_r | GACAGAGTGAGAGAGCAATAGTACAGAGA |
| hsp70+58_f | CAATTCAAACAAGCAAAGTGAACAC |
| hsp70+58_r | TGATTCACTTTAACTTGCACTTTA |
| hsp70+681_f | CACCACGCCGTCCTACGT |
| hsp70+681_r | GGTTCATGGCCACCTGGTT |
| hsp70+1427_f | CTGTGCAGGCCGCTATCC |
| hsp70+1427_r | GCGCTCGATCAGCTTGGT |
| hsp70+1702_f | GGGTGTGCCCCAGATAGAAG |
| hsp70+1702_f | TGTCGTTCTTGATCGTGATGTTC |
| hsp70+2065_f | AGGAGCTCACCCGCCACT |
| hsp70+2065_r | CTGCTGGCCGCAGTTTGCT |
| hsp70-2549_f | GTTGGCATCCCTATTAAACAGC |
| hsp70-2549_r | CAGGACTCACTTAGCGGGG |
| intergenic_f | TGCTGACTGCCATCAAATTC |
| intergenic_r | TACTGCTGTGACGGCTTTG |
