# Supplementary material for: Stress-Induced PARP Activation Mediates Recruitment of Drosophila Mi-2 to Promote Heat Shock Gene Expression
Source: PLoS Genet. 2011 Jul 28;7(7):e1002206. doi: 10.1371/journal.pgen.1002206 (PMC3145624; doi:10.1371/journal.pgen.1002206)
Supplement: Dataset S3 — Sequences of primers used for RT-QPCR. (DOCX) [file pgen.1002206.s003.docx]

RT-QPCR pimers:

| Primer name | Sequence (from 5´ to 3´) |
| --- | --- |
| rp49_f | CGGATCGATATGCTAAGCTG |
| rp49_r | GAACGCAGGCGACCGTTGGGG |
| actin5C_f | AAGTTGCTGCTCTGGTTGTCG |
| actin5C_r | GCCACACGCAGCTCATTGTAG |
| GAPDH_f | GAGCAAGGACTAAACTAGCCAAA |
| GAPDH_r | CAACAGTGATTCCCGACCA |
| hsp70_f | ATATCTGGGCGAGAGCATCACA |
| hsp70_r | GTAGCCTGGCGCTGGGAGTC |
| hsp70total_f | TTGGGCGGCGAGGACTTTG |
| hsp70total_r | GCTGTTCTGAGGCGTCGTAGG |
| hsp70-3end_f | GTTGGCATCCCTATTAAACAGC |
| hsp70-3end_r | CAGGACTCACTTAGCGGGG |
| hsp26_f | CACCGTCAGTATTCCCAAGC |
| hsp26_r | GTTCTCCTTGCCCTTCACC |
| hsp83_f | TCTGTGAATAGAACGAAAAATACA |
| hsp83_r | TGATGATCAGGGACATCAGC |
| hsp83total_f | GGGTTTCTACTCCGCCTACC |
| hsp83total_r | CACGTACTGCTCGTCATCGT |
| hsp83-3end_f | GATGACCCGATCGATGATAAA |
| hsp83-3end_r | CCCCCAATAAATACTCGCTCA |
| hsp83-intr_f | TCCTTAGTGTTGAACCCACAGA |
| hsp83-intr_r | TCTCTGCTTCTTCTGGCATC |
