# Supplementary material for: Stress-Induced PARP Activation Mediates Recruitment of Drosophila Mi-2 to Promote Heat Shock Gene Expression
Source: PLoS Genet. 2011 Jul 28;7(7):e1002206. doi: 10.1371/journal.pgen.1002206 (PMC3145624; doi:10.1371/journal.pgen.1002206)
Supplement: Dataset S4 — Sequences of primers used for ds RNA synthesis (RNAi). (DOCX) [file pgen.1002206.s004.docx]

RNAi QPCR pimers (for dsRNA synthesis)

| **Oligoname** | **Sequence** |
| --- | --- |
| T7-dMi-2_f | TAATACGACTCACTATAGGGTTAACTCGCTGACCAAGGCT |
| T7-dMi-2_r | TAATACGACTCACTATAGGGATATCGTTGTGGGGATTCCA |
| T7-Luc_f | TAATACGACTCACTATAGGGGGAAGAACGCCAAAAAC |
| T7-Luc_r | TAATACGACTCACTATAGGGCTCTGGCACAAAATCG |
