# Supplementary figures and images for: Stress-Induced PARP Activation Mediates Recruitment of Drosophila Mi-2 to Promote Heat Shock Gene Expression
Source: PLoS Genet. 2011 Jul 28;7(7):e1002206. doi: 10.1371/journal.pgen.1002206 (PMC3145624; doi:10.1371/journal.pgen.1002206)

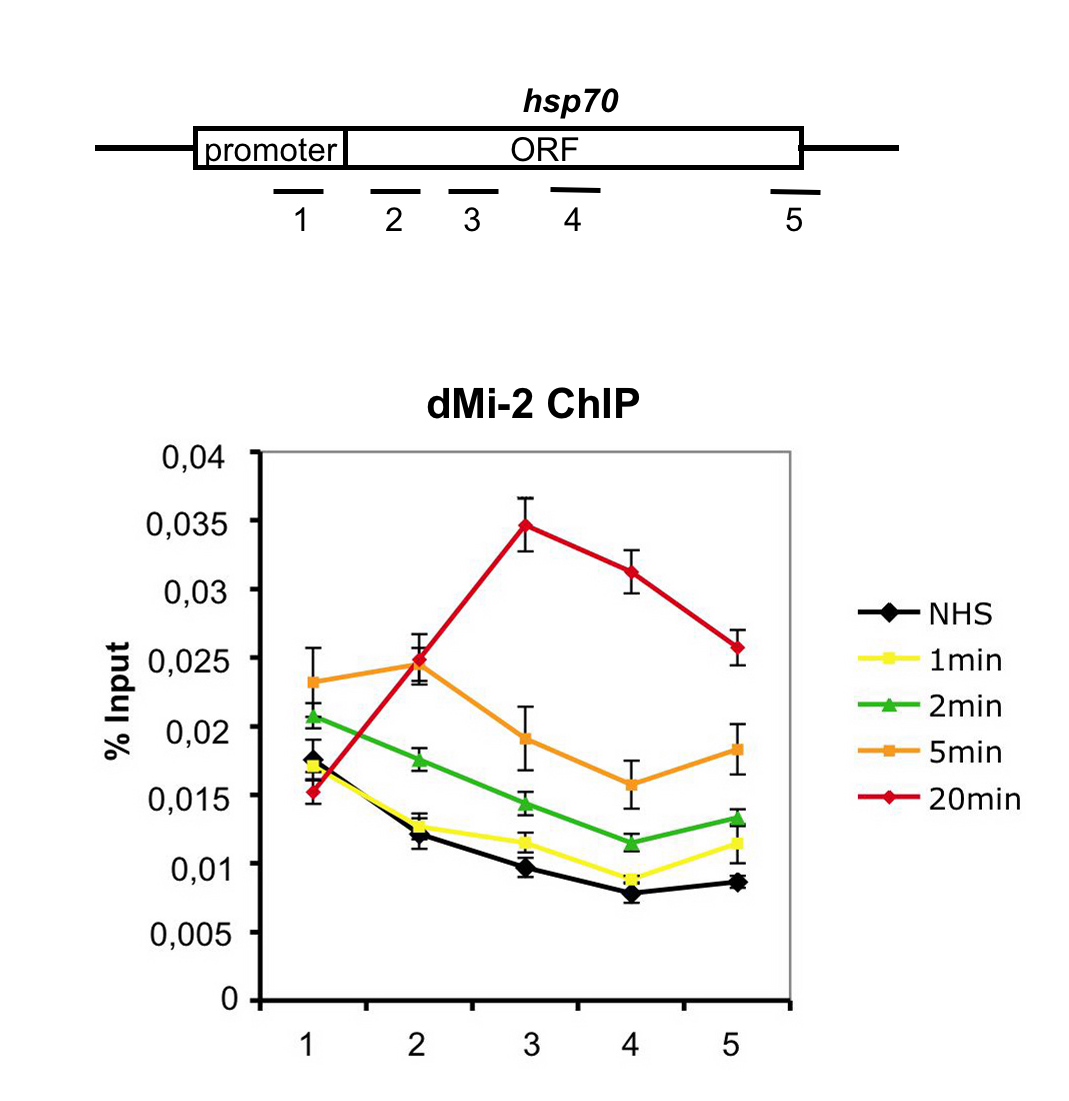

Supplement: Figure S1 — Kinetic analysis of dMi-2 binding to hsp70 gene during heat shock. dMi-2 binding to hsp70 gene was determined by ChIP under NHS conditions and at different time points following heat shock as indicated. Amplimers were centered as follows: 1, -154 bp; 2, +58 bp; 3, +681 bp; 4, +1427 bp; 5, + 2549 bp. (TIF) [file pgen.1002206.s005.tif]

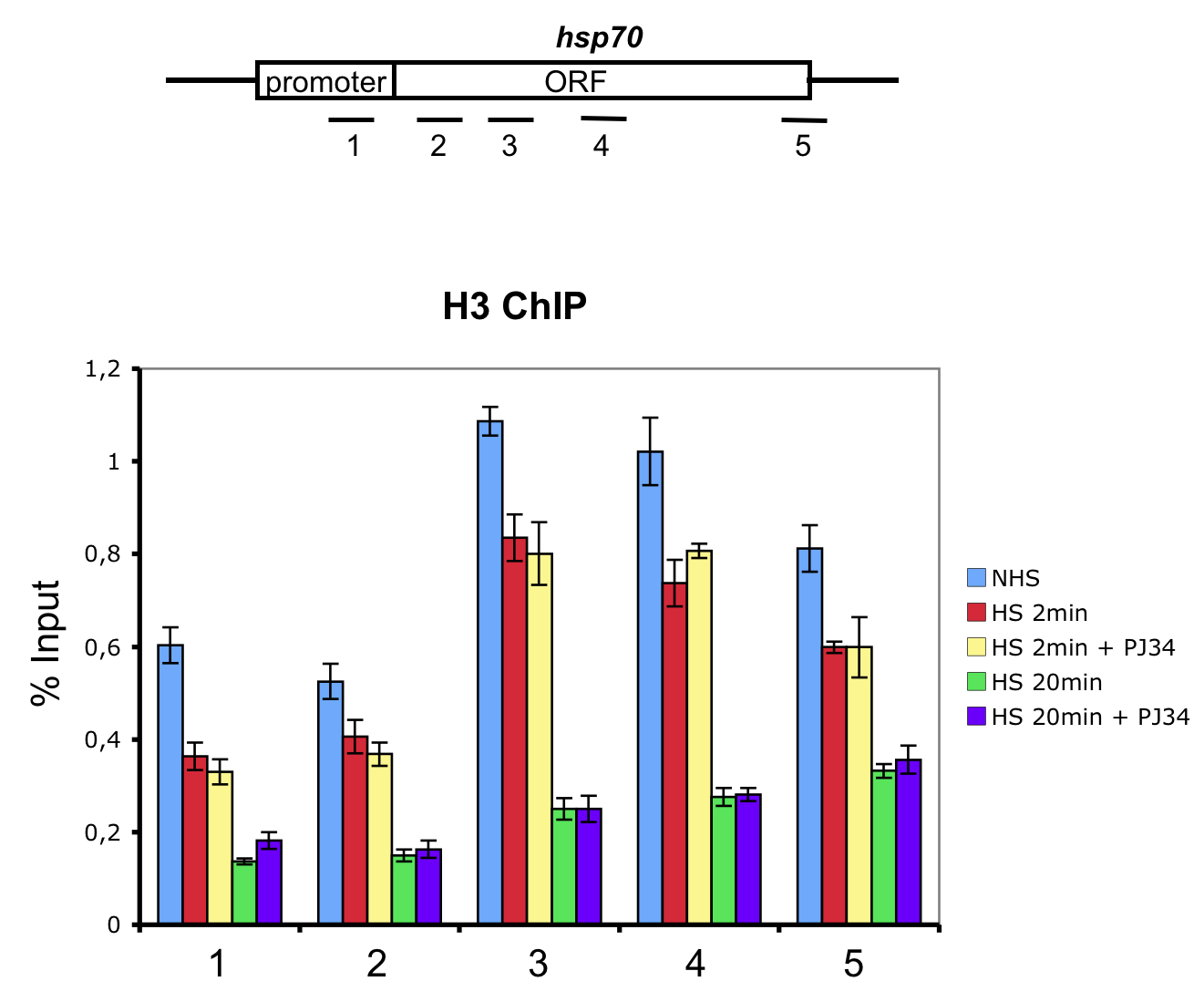

Supplement: Figure S2 — H3 ChIP on hsp70 gene upon PJ34 treatment. dMi-2 binding to hsp70 gene was determined by ChIP under NHS conditions and at different time points in the absence or in the presence of PJ34, as indicated. Amplimers were centered as follows: 1, -154 bp; 2, +58 bp; 3, +681 bp; 4, +1427 bp; 5, + 2549 bp. (TIF) [file pgen.1002206.s006.tif]

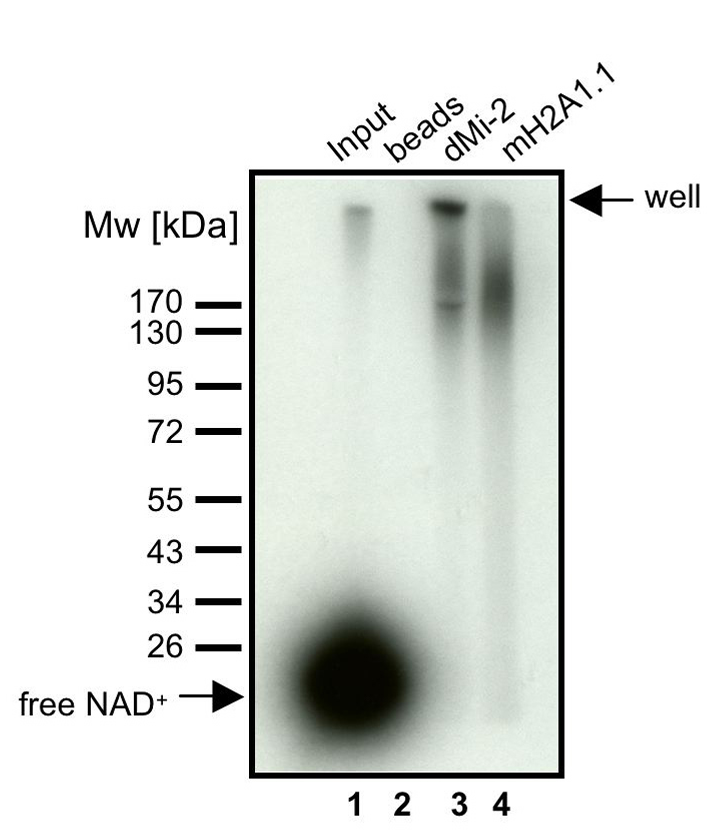

Supplement: Figure S3 — Pulldown with whole PAR reaction. Experiment was performed as in Figure 3A with a difference that radioactive NAD+ was used for PAR synthesis. Samples were run on the gel, gel was dried and exposed overnight on the X-ray film. (TIF) [file pgen.1002206.s007.tif]

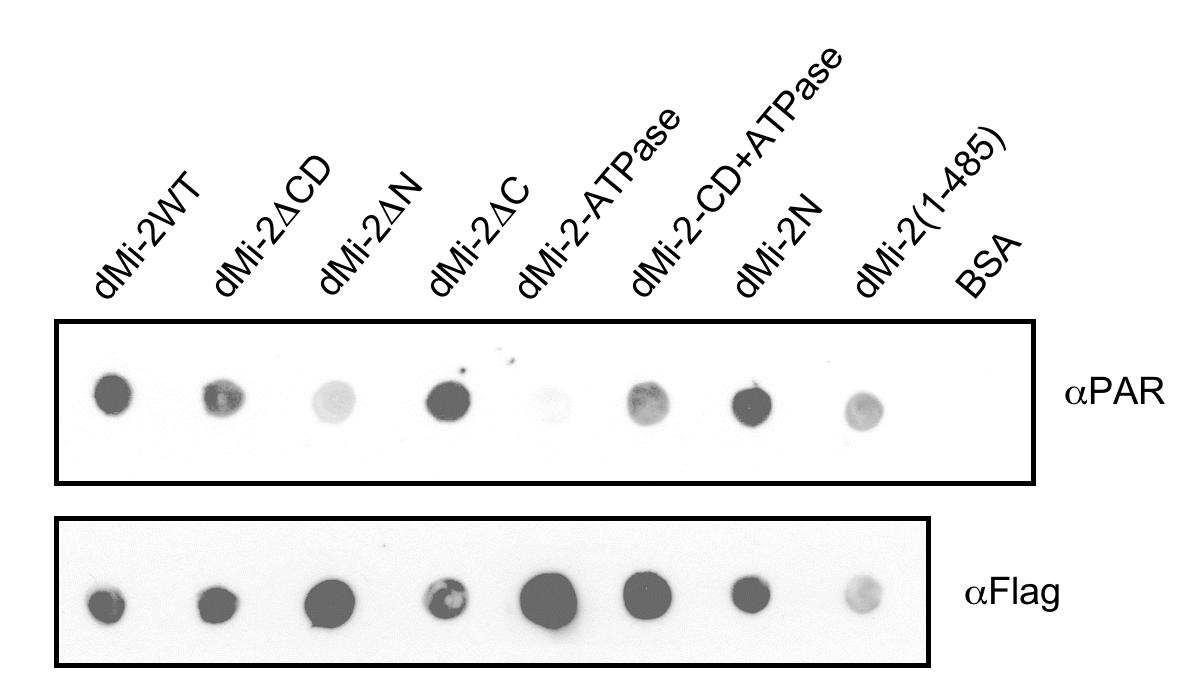

Supplement: Figure S4 — PAR binding assay. Dot blot with purified PAR. BSA or recombinant dMi-2 WT and indicated mutants were spotted on the nitrocellulose and incubated with PAR. Upon extensive washes, membrane was subjected to Western Blot analysis with anti-PAR antibodies. After stripping, membrane was probed with anti-Flag antibodies to monitor the amount of proteins spotted. (TIF) [file pgen.1002206.s008.tif]

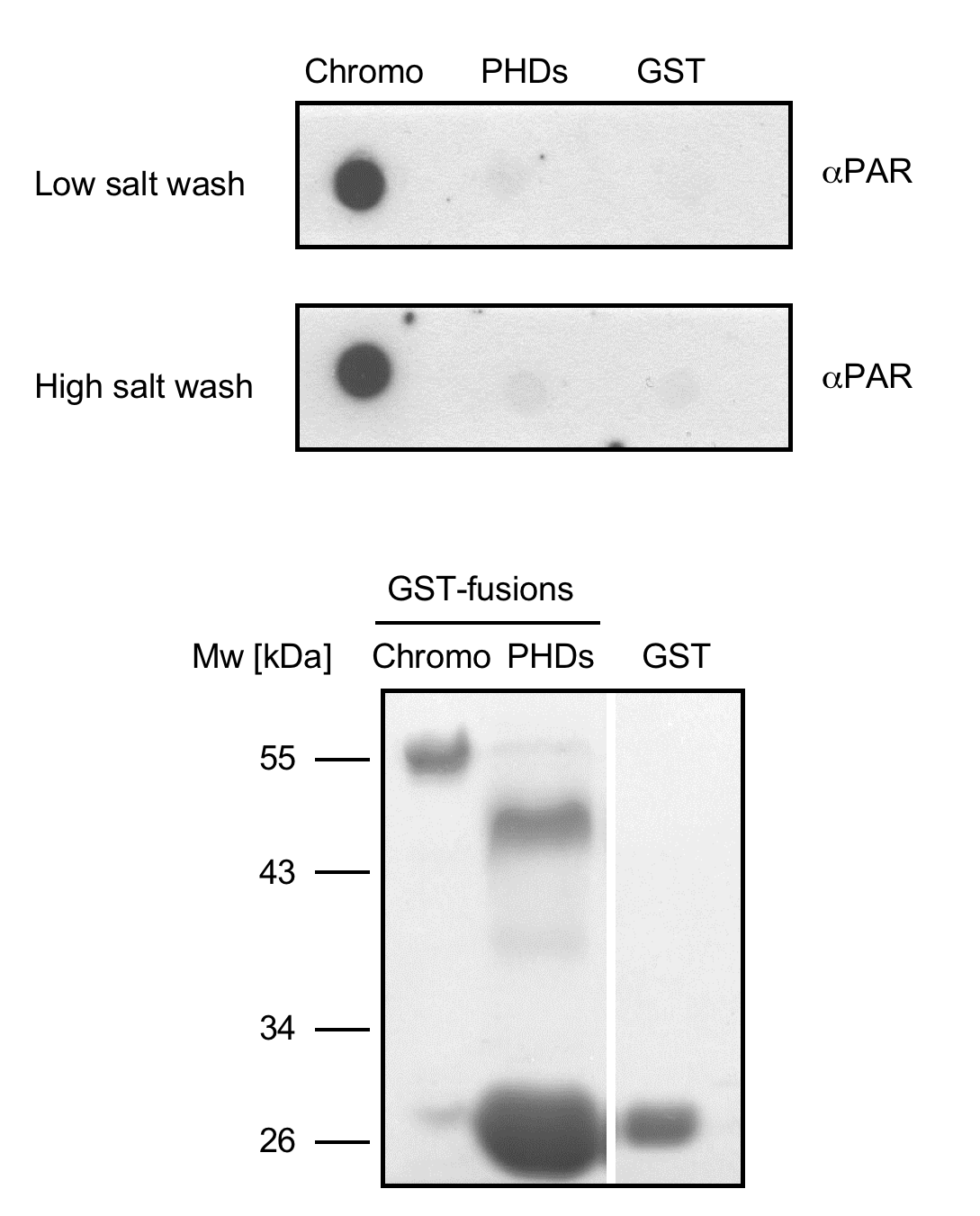

Supplement: Figure S5 — PAR binding assay. Upper panel: Dot blot with purified PAR. GST-fusion proteins and GST were spotted on the nitrocellulose and incubated with PAR. Upon extensive washes with low salt (150 mM) or high salt (500 mM), membranes were subjected to Western Blot analysis with anti-PAR antibodies. Lower panel: Coomasie stained gel with purified proteins used for PAR binding assay. Chromo - chromodomains of dMi-2 (aa 488-712), PHDs – PHD fingers of dMi-2 (aa 377-490). (TIF) [file pgen.1002206.s009.tif]

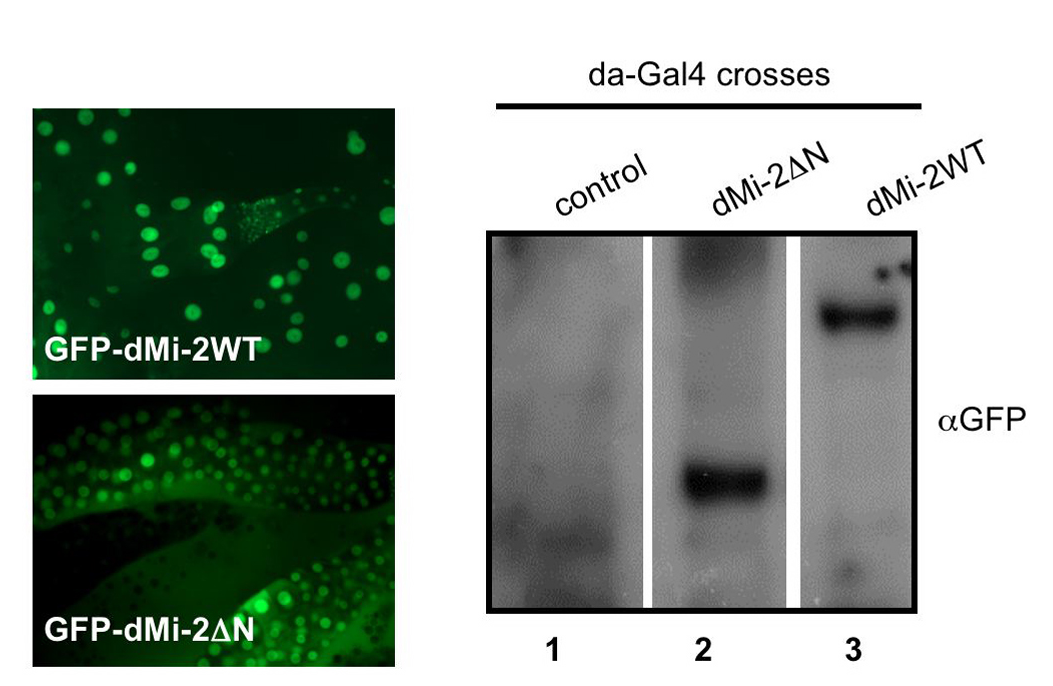

Supplement: Figure S6 — Expression analysis of GFP-tagged transgenes. Left panel – whole salivary glands from flies crossed to the salivary gland-specific sgs58ABGAL4 driver were analysed for GFP expression. Right panel: larval extracts derived from control (w1118) larvae (line 1) and larvae expressing GFPtagged dMi-2WT (lane 3) or dMi-2ΔN transgene (lane 2) crossed to daughterless-GAL4 driver were analysed by western blot using GFP antibodies. (TIF) [file pgen.1002206.s010.tif]

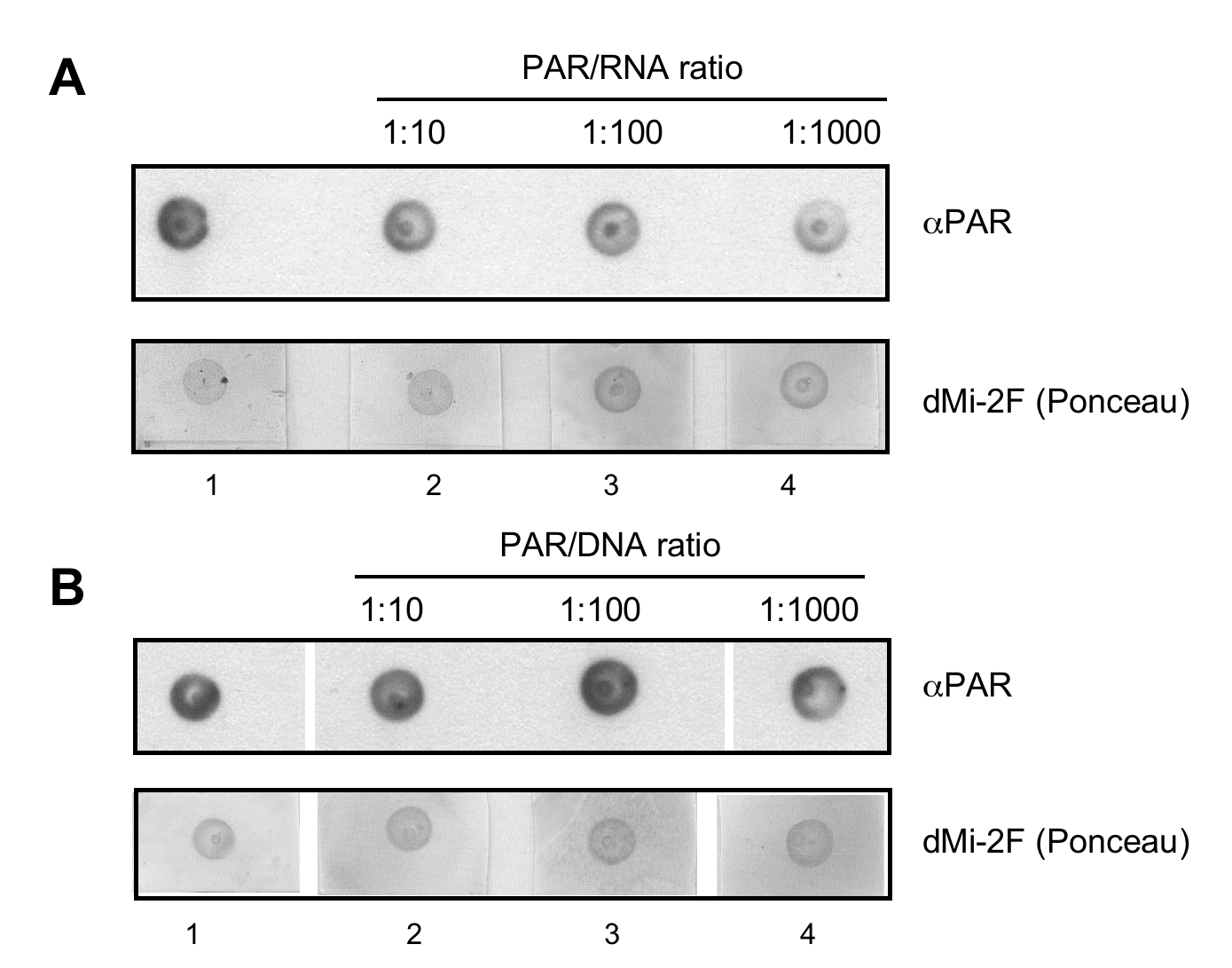

Supplement: Figure S7 — Competition of PAR binding with RNA and DNA. (A) Dot blot with purified PAR. dMi-2 WT was spotted on the nitrocellulose and incubated with PAR. Upon extensive washes membranes were subjected to Western Blot analysis with anti-PAR antibodies (upper panel), Ponceau staining indicates the amount of protein spotted (lower panel). When indicated, membranes were preincubated with increasing amounts of RNA (lanes: 2,3 and 4) followed by incubation with PAR. Lane 1: dMi-2 was preincubated with buffer only. (A) Dot blot with purified PAR. dMi-2 WT was spotted on the nitrocellulose and incubated with PAR. When indicated, membranes were preincubated with increasing amounts of DNA (lanes: 2,3 and 4) followed by incubation with PAR. Lane 1: dMi-2 was preincubated with buffer only. (TIF) [file pgen.1002206.s011.tif]

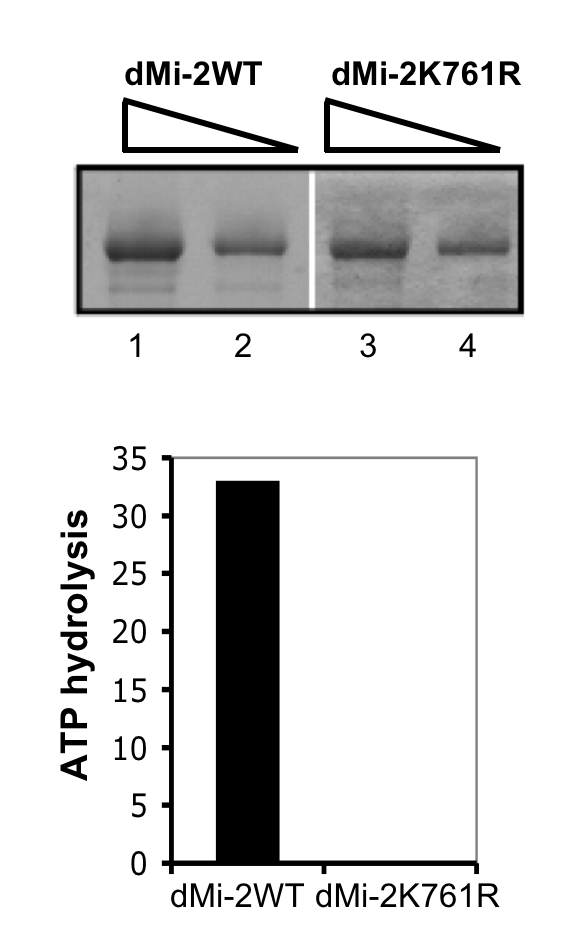

Supplement: Figure S8 — dMi-2 K761R mutant is catalytically inactive. Upper panel: a Coomasie gel with dMi-2 WT and dMi-2 K761R mutant. Lower panel: ATPase assay with wild type and mutant form of dMi-2 in the presence of nucleosomes. (TIF) [file pgen.1002206.s012.tif]
