# Supplementary material for: Stress-Induced PARP Activation Mediates Recruitment of Drosophila Mi-2 to Promote Heat Shock Gene Expression
Source: PLoS Genet. 2011 Jul 28;7(7):e1002206. doi: 10.1371/journal.pgen.1002206 (PMC3145624; doi:10.1371/journal.pgen.1002206)
Supplement: Text S1 — Supporting protocols: Fly strains, transgenesis and dot blot assay. (DOC) [file pgen.1002206.s013.doc]

**Supporting Protocols:**

**Fly strains and transgenesis**

The pUASTattB vector was used for generation of expression constructs. dMi-2 WT and dMi-2 K671R inserts were created by PCR and site directed

mutagenesis and ligated into pUASTattB following standard procedures. For generation of GFP constructs, the pUASTattBeGFP vector was constructred by inserting eGFP coding sequence into pUASTattB. dMi-2 WT and dMi-2DN were amplified with PCR and ligated into pUASTattBeGFP. All constructs were confirmed by sequencing. For generation of transgenic flies the C31 attP/attB system was used. The j5 strain (attP-zh86Fb/vas-phi-zh102D) with an attP landing site on the third chromosome (kind gift from K. Basler) was used for injections. F0 flies were crossed to the w1118 isogenic strain (BL # 5905), the F1 generation was screened for orange eyes and then crossed against each other to obtain homozygotic UAS strains. The following UAS strains were obtained: dMi-2_Flag, dMi-2K671R_Flag, GFPdMi-2, GFPdMi-2DN, GFP. For dMi-2 knockdown an dMi-2 RNAi strain was used (VDRC, # 107204/GD). For overexpression or knockdown experiments the following Gal4-driver strains

were used: da-Gal4, sgs14.2-Gal4, sgs58AB-Gal4.

**Dot blot assay**

Poly(ADP-ribose) was purified as previously described {Karras, 2005 #45}.

Recombinant flag-tagged proteins were purified from baculovirus infected SF9 cells and eluted with Flag peptide. Nitrocellulose membrane was equilibrated in TBS-T buffer (10 mM Tris, pH 7.4, 0,15M NaCl, 0,05% Tween20). 0,4 μg of dMi-2 WT or dMi-2 mutants or 1 μg of BSA or 1 μg of GST-fusion proteins were spotted on the membrane and air-dried. The membrane was incubated with purified PAR (200 nM) for 2,5 hrs at RT with gentle agitation. For competition assays, membranes with spotted dMi-2 WT were preincubated with the increasing amounts of RNA (hsp70 fragment used for band shift assays) or DNA (pUC vector) for 1,5 hr before PAR solution as added. The membranes were incubated for another 1,5 hrs followed by extensive washing with TBS-T. Washed membranes were subjected to Western blot analysis with anti-PAR antibodies.
